# Advances in drug delivery systems for the management of gout and hyperuricemia

**DOI:** 10.3389/fphar.2025.1660890

**Published:** 2025-10-01

**Authors:** Zining Peng, Fanyu Meng, Qian Deng, Yuanbo Huang, Danning Mao, Yuan Long, Weitian Yan, Jiangyun Peng, Xingqiang Wang, Nian Liu

**Affiliations:** ^1^ The First School of Clinical Medicine, Yunnan University of Chinese Medicine, Kunming, China; ^2^ Department of Rheumatology, The No.1 Affiliated Hospital of Yunnan University of Chinese Medicine/Yunnan Provincial Hospital of Traditional Chinese Medicine, Kunming, China; ^3^ Yunnan Key Laboratory of Southern Medicinal Utilization, Yunnan University of Chinese Medicine, Kunming, China; ^4^ Yunnan Provincial Clinical Medicine Research Center of Rheumatism in TCM, Yunnan Provincial Hospital of Traditional Chinese Medicine, Kunming, China

**Keywords:** gout, hyperuricemia, drug delivery systems, therapeutic, prospect

## Abstract

Gout and hyperuricemia represent significant global health burdens, characterized by painful inflammatory arthritis and systemic metabolic dysfunction, respectively. Current pharmacological management faces substantial limitations, including poor bioavailability, systemic toxicity, narrow therapeutic indices, immunogenicity, and suboptimal patient adherence due to frequent dosing and adverse effects. These challenges underscore the critical need for innovative therapeutic strategies. Advanced drug delivery systems (DDSs) have emerged as transformative solutions to overcome these hurdles. This comprehensive review critically analyzes recent advances in DDSs tailored to the management of gout and hyperuricemia. We first elucidate the intricate pathophysiological mechanisms linking hyperuricemia, monosodium urate (MSU) crystal deposition, NLRP3 inflammasome activation, and chronic inflammation. We then systematically explore cutting-edge DDS platforms, including lipid-based, polymer-based, and other systems. These engineered drug delivery systems significantly enhance therapeutic outcomes in gout and hyperuricemia by improving drug solubility, enabling targeted delivery, providing sustained release, facilitating synergistic drug co-delivery, and responding to pathological microenvironments, although preclinical evidence is limited and clinical evidence supporting their efficacy and safety remains sparse. Finally, we highlight translational challenges and future directions while emphasizing the considerable promise of integrating AI, biomaterial science, and personalized medicine to advance patient-centric DDS. Although progress has been made, sustained interdisciplinary collaboration and rigorous clinical validation remain critical to translate these innovations into tangible improvements in long-term disease management and quality of life for patients with gout and hyperuricemia.

## 1 Introduction

Hyperuricemia, defined as elevated serum urate (SU) levels (>6.8 mg/dL), is a metabolic disorder affecting approximately 20% of the global population, with its prevalence increasing due to dietary changes, obesity, and aging populations (Nakayama et al., 2023; Zhong et al., 2024). Gout, the most common inflammatory arthritis worldwide, arises from the deposition of monosodium urate (MSU) crystals in joints and soft tissues, driven by chronic hyperuricemia (Cabau et al., 2023; Nakayama et al., 2023). Epidemiological studies highlight a strong association between hyperuricemia/gout and comorbidities such as cardiovascular diseases, chronic kidney disease, and metabolic syndrome, further amplifying their clinical and socioeconomic burden (Cabau et al., 2023; Nakayama et al., 2023). Genetic predispositions, including mutations in urate transporters (e.g., URAT1), also contribute to disease susceptibility (Sun et al., 2024). Alarmingly, the global incidence of gout has increased significantly in recent decades, particularly in the Western and Asia–Pacific regions, underscoring the need for improved management strategies (Nakayama et al., 2023; Zhong et al., 2024; Dalbeth et al., 2021).

Current clinical guidelines for hyperuricemia and gout emphasize a dual approach: (1) acute flare management using nonsteroidal anti-inflammatory drugs (NSAIDs), colchicine, or corticosteroids and (2) long-term urate-lowering therapy (ULT) with xanthine oxidase inhibitors (e.g., allopurinol and febuxostat) or uricosurics (e.g., probenecid) (Karantas et al., 2024; Sattui and Gaffo, 2016). Despite these options, significant limitations persist. Allopurinol, the first-line ULT, suffers from reduced efficacy in patients with impaired conversion to its active metabolite, oxypurinol (OXY), and carries risks of severe hypersensitivity reactions (Mohamad et al., 2025; Yi et al., 2024). Oral and injectable formulations of existing drugs often exhibit poor bioavailability, systemic toxicity, and suboptimal patient adherence due to frequent dosing and adverse effects (e.g., gastrointestinal distress and rashes) (Sattui and Gaffo, 2016). For instance, the narrow therapeutic index of colchicine necessitates cautious dosing to prevent toxicity, while uricase therapies (e.g., pegloticase) are challenged by immunogenicity and instability (Cabau et al., 2023; Yang Y. et al., 2023).

Moreover, traditional drug delivery systems (DDSs)—primarily oral tablets and intravenous injections—fail to address localized MSU crystal deposition or provide sustained drug release, leading to recurrent flares and chronic joint damage (Mohamad et al., 2025; Yi et al., 2024). The lack of targeted delivery exacerbates off-target effects and limits therapeutic efficacy, particularly in patients with comorbidities requiring polypharmacy (Karantas et al., 2024). These shortcomings highlight the critical need for innovative DDSs to enhance drug safety, bioavailability, and patient compliance (Ezike et al., 2023).

Advanced DDSs offer transformative solutions to overcome the limitations of conventional therapies. By leveraging nanotechnology, biomaterials, and controlled-release mechanisms, modern DDSs provide the following therapeutic advantages. First, enhanced bioavailability: nanocarriers (e.g., liposomes and polymeric nanoparticles) improve solubility and stability of poorly water-soluble drugs like OXY and colchicine, ensuring consistent therapeutic plasma levels (Peng et al., 2023; Singh et al., 2017). Second, targeted delivery: ligand-functionalized carriers selectively accumulate drugs in inflamed joints or renal tissues, minimizing systemic exposure and adverse effects (Ezike et al., 2023; Yang B. et al., 2023). For example, transdermal microneedles loaded with allopurinol bypass hepatic metabolism, reducing first-pass effects and enabling sustained release (Chen et al., 2021). Third, sustained and controlled release: hydrogels and 3D-printed porous scaffolds (e.g., tantalum-based systems) provide localized, long-term drug delivery, ideal for managing chronic gout and preventing crystal recurrence (Hua et al., 2021; Wang et al., 2025). Fourth, combination therapy: co-delivery systems (e.g., lipid-based vesicles co-encapsulating anti-inflammatory and urate-lowering agents) synergistically address inflammation and hyperuricemia, simplifying treatment regimens (Li et al., 2023).

Recent advancements in DDSs have yielded promising preclinical and clinical outcomes (Jafernik et al., 2023). Transdermal and topical systems: ethosomes and transfersomes loaded with capsaicin (CAP) and triamcinolone acetonide (TCS) enhance skin permeation, offering localized analgesia and anti-inflammatory effects in acute gout (Singh et al., 2017). Similarly, dissolving microneedles (DMNs) for allopurinol delivery demonstrate sustained SU reduction in animal models, avoiding gastrointestinal toxicity (Singh et al., 2017). Nanoformulations: nonionic surfactant-based niosomes encapsulating allopurinol achieved 82% drug release within 24 h and significantly reduced SU levels in MSU crystal-induced gout models, outperforming free drug formulations (Singh et al., 2017). Lipid–polymer hybrid nanoparticles and inorganic carriers (e.g., silica nanoparticles) also improve uricase stability and targeting (Wang et al., 2025). Smart and responsive systems: pH-sensitive hydrogels and enzyme-responsive nanocapsules release drugs selectively in acidic or inflamed microenvironments, enhancing precision (Wang et al., 2025; Liu et al., 2024). For instance, a colchicine-coordinated nanogel system reduced the dosing frequency and mitigated systemic toxicity in chronic gout models (Yang B. et al., 2023). Biological and biomimetic systems: erythrocyte membrane-coated nanoparticles and macrophage-derived exosomes exploit natural trafficking mechanisms to deliver drugs to synovial tissues, demonstrating reduced immunogenicity (Wang et al., 2025).

This review presents a synthesized perspective on the pathophysiological mechanisms and therapeutic strategies for gout and hyperuricemia, with a nuanced focus on delineating the evolving role of DDS. While acknowledging the current reliance on preclinical studies and limited clinical validation data, this work underscores the promising potential of DDS-driven approaches to advance precision medicine frameworks. By integrating cutting-edge nanomedicine approaches, we critically analyze the molecular targets underpinning urate crystal deposition, NLRP3 inflammasome activation, and chronic inflammatory cascades in gout while contextualizing hyperuricemia as a modifiable risk factor. The manuscript highlights how DDSs—including stimuli-responsive nanoparticles, targeted ligands, and biodegradable carriers—enhance solubility, bioavailability, and tissue-specific delivery of urate-lowering agents (e.g., allopurinol and febuxostat) and anti-inflammatory therapies (e.g., colchicine and IL-1β inhibitors) (Chen Z. et al., 2025; Liu S. et al., 2023). By integrating mechanistic discoveries with emerging therapeutic innovations, the analysis highlights how DDS research is laying critical groundwork for future translational efforts. Although widespread clinical implementation awaits further validation through large-scale trials, these findings offer valuable insights to guide early-stage translational research and inform evidence-based decision-making in the evolving landscape of gout and hyperuricemia management.

## 2 Pathogenesis and pathophysiology of gout and hyperuricemia

### 2.1 Pathogenesis and pathophysiology of gout

Gout, a prototypical disorder of metabolic–immune dysregulation, is an inflammatory arthritis triggered by the deposition of MSU crystals. Its pathophysiology centers on chronic hyperuricemia and dysregulated uric acid metabolism (Bhadouria et al., 2025; Stiburkova and Ichida, 2025; Zhao et al., 2022). This section elaborates on its pathogenesis from multiple perspectives, including molecular mechanisms, inflammatory responses, genetic factors, and environmental regulation.

#### 2.1.1 MSU crystal deposition and inflammatory activation

**FIGURE 1:**
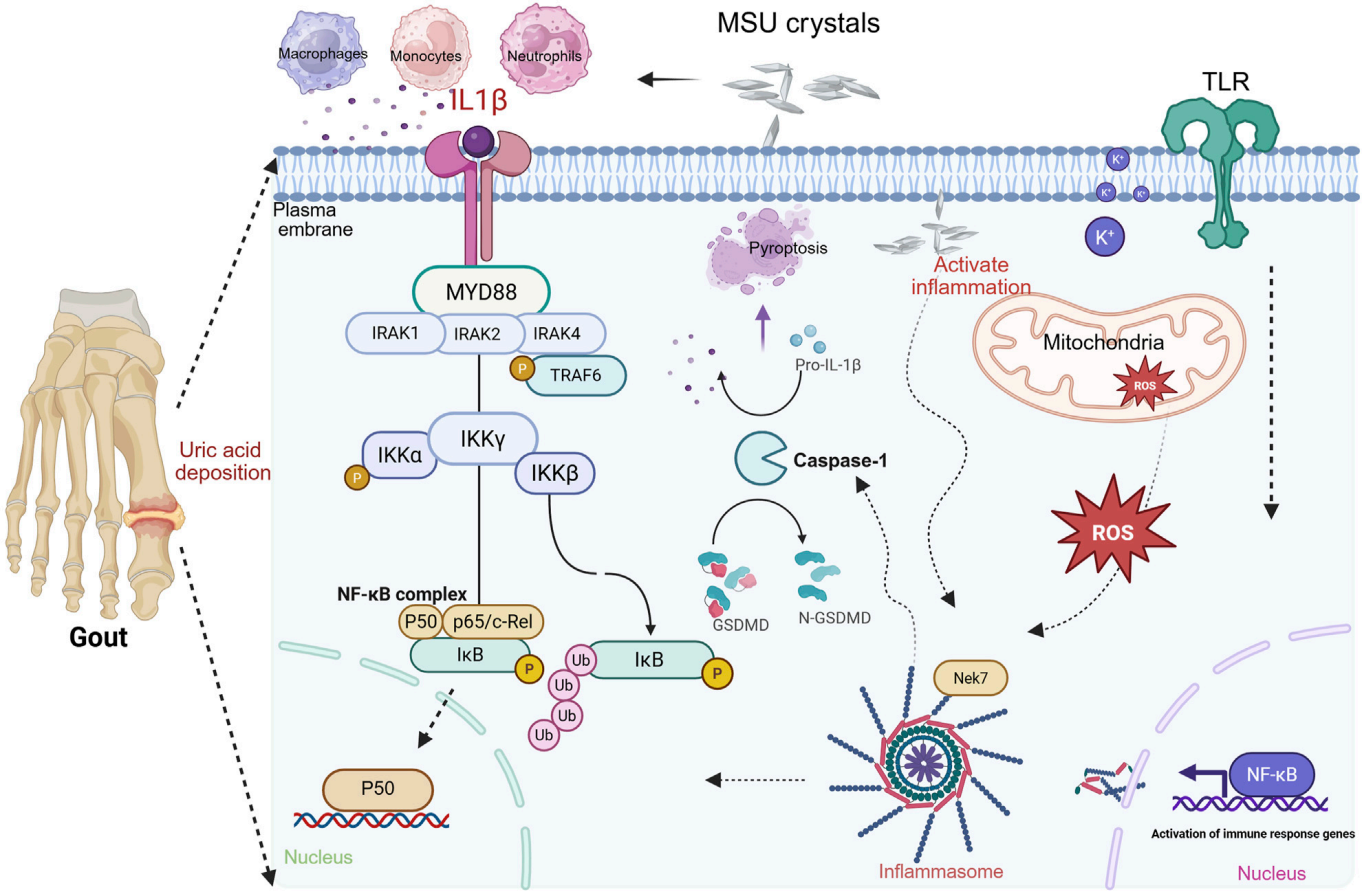
Integrated mechanism of NLRP3 inflammasome activation and IL-1 signaling synergy in driving inflammatory cascades. Note: NLRP3 inflammasome activation occurs via a dual-signal mechanism. Signal 1 (Priming): NF-κB-dependent pathways (e.g., Toll-like receptor activation) induce expressions of NLRP3 and pro-IL-1β. Signal 2 (Activation): MSU crystals trigger NLRP3 oligomerization through K^+^ efflux, mitochondrial dysfunction, and ROS release. MSU interaction recruits Nek7 kinase, binding NLRP3 to drive inflammasome assembly. Cleavage of gasdermin D, generating pore-forming fragments that induce pyroptosis and inflammatory mediator release. Secreted IL-1β recruits MyD88, IRAK1/2/4, and TRAF6. This activates the IKK complex, leading to NF-κB nuclear translocation and transcription of chemokines/cytokines, establishing a positive feedback loop that amplifies inflammation (created in BioRender. (2025) https://BioRender.com/kp5tvi0).

The direct trigger of gout is the deposition of MSU crystals within joints and periarticular tissues. MSU crystal formation follows a three-stage kinetic model: nucleation, growth, and aggregation. When serum uric acid (SUA) concentration exceeds 6.8 mg/dL (its saturation solubility), urate precipitates from supersaturated body fluids to form crystals (Bhadouria et al., 2025; Narang and Dalbeth, 2020). Following deposition, these crystals trigger an inflammatory cascade by activating the innate immune system (e.g., macrophages and neutrophils). MSU crystals activate innate immunity via two primary modes. Pattern recognition receptor (PRR)-dependent pathway: cholesterol-rich microdomains exposed on the crystal surface directly bind to Toll-like receptors 2/4 (TLR2/4), thereby initiating the MyD88 signaling cascade (Bhadouria et al., 2025; Narang and Dalbeth, 2020). This leads to NF-κB-mediated expression of pro-inflammatory cytokines (IL-6 and TNF-α), constituting a core mechanism of acute gout attacks. Lysosomal damage pathway: Upon phagocytosis by macrophages, crystals cause lysosomal membrane rupture, releasing cathepsin B, which activates the NLRP3 inflammasome (Kluck et al., 2021; Zhang, 2023). This complex cleaves pro-IL-1β via caspase-1 to generate active IL-1β (a key inflammatory mediator), concurrently inducing gasdermin D-mediated pyroptosis. The integration mechanism of NLRP3 inflammasome activation and the IL-1 signaling pathway, synergistically driving the inflammatory cascade response, is shown in Figure 1. This integrated model reveals the regulatory network from NLRP3 inflammasome activation and effector release to IL-1-mediated amplification of inflammatory cascades.

#### 2.1.2 Genetic predisposition

GWASs have identified multiple gout-associated genetic loci, revealing a dual-pathway “urate metabolism-inflammatory regulation” genetic architecture. This includes urate transporter genes (e.g., *ABCG2*, *URAT1/SLC22A12*, and *GLUT9/SLC2A9*), primarily associated with impaired intestinal/renal uric acid excretion.

Inflammatory regulator genes (e.g., *IL-1β* and *IL-6*): primarily linked to inflammasome hyperactivation (Chen et al., 2018; Kawaguchi et al., 2021; Nakayama et al., 2023; Eckenstaler and Benndorf, 2021). Metabolic interaction module genes (e.g., *GCKR* and *PDZK1*): involved in dysregulated glucose/lipid metabolism impacting urate homeostasis. Notably, loss-of-function mutations in ABCG2 (e.g., Q141K) impair intestinal urate excretion, thereby significantly increasing the risk of gout (Kawaguchi et al., 2021; Eckenstaler and Benndorf, 2021; Dong et al., 2020). Furthermore, epistasis between PKD2 and ABCG2 further influences serum urate levels, highlighting the importance of polygenic synergy in gout pathogenesis (Dong et al., 2020).

#### 2.1.3 Epigenetic reprogramming

Recent studies have revealed distinct DNA methylation patterns in patients with gout compared to those with hyperuricemic individuals. For instance, the promoter regions of inflammation-related genes (e.g., IL-6 and TNF-α) in patients with gouty arthritis exhibit hypomethylation, potentially promoting inflammation directly and independently of hyperuricemia (Tseng et al., 2020). Additionally, dynamic regulation of histone modifications (e.g., H3K27ac) plays a role. MSU crystals activate the p300/CBP acetyltransferase, inducing H3K27ac enrichment at enhancer regions of inflammatory genes (e.g., *CXCL8* and *PTGS2*) within macrophages, thereby prolonging inflammatory responses (Punzi et al., 2025).

#### 2.1.4 Impaired intestinal and renal excretion

Approximately 90% of patients with gout exhibit impaired uric acid excretion, primarily due to reduced renal urate clearance (Abrahim, 2021). Renal excretion: URAT1 (SLC22A12) is the key urate reabsorption transporter in the renal proximal tubule. Its enhanced function or upregulated expression contributes to hyperuricemia (Nakayama et al., 2023; Abrahim, 2021). Proximal tubular epithelial cells reabsorb urate through coordinated transport mediated by URAT1 (SLC22A12) and GLUT9 (SLC2A9). The URAT1 inhibitor benzbromarone reduces its Vmax, while the SLC2A9 eQTL variant (rs7442295) decreases the fractional excretion of uric acid. Intestinal excretion: ABCG2-mediated intestinal urate excretion accounts for ∼30% of total elimination. ABCG2 dysfunction can induce “secondary hyperuricemia.” Gut microbiota metabolites (e.g., butyrate) upregulate ABCG2 expression by activating PPARγ, suggesting therapeutic potential via microbe–host interaction (Eckenstaler and Benndorf, 2021).

#### 2.1.5 Synergism with metabolic syndrome

Gout is closely associated with metabolic syndrome components, including obesity, insulin resistance, and hypertension (Abrahim, 2021; Pascart and Liote, 2019). An imbalance in adipose tissue-derived adipokines (leptin and adiponectin) may exacerbate gout by inhibiting renal urate excretion and promoting inflammation. An elevated leptin/adiponectin ratio suppresses AMPK phosphorylation, downregulates renal tubular ABCG2 expression, and activates the NLRP3 inflammasome, creating a “metabolic inflammation” microenvironment (Pascart and Liote, 2019; Gong et al., 2020). Furthermore, high fructose intake activates hepatic fructokinase, accelerating ATP degradation into uric acid precursors while inducing insulin resistance. Fructokinase catalyzes fructose conversion to fructose-1-phosphate, consuming ATP to generate AMP, which is subsequently converted to uric acid via the xanthine oxidase (XO) pathway. A single fructose load (>50 g) can increase SUA by 1.5 mg/dL for up to 6 h, establishing a vicious cycle (Pascart and Liote, 2019; Abrahim, 2021).

#### 2.1.6 Transition from asymptomatic hyperuricemia to gout

Only 10%–20% of individuals with hyperuricemia develop gout, implicating critical roles for other regulatory factors (e.g., local tissue microenvironment and crystal-promoting factors) (Ohashi et al., 2024; Zhang, 2023). Studies have shown that asymptomatic individuals with hyperuricemic already exhibit subclinical inflammation (e.g., elevated IL-6 and CRP), whereas acute gout flares are characterized by significant increases in IL-1β and S100A8/A9 levels, which may serve as distinguishing biomarkers (Ohashi et al., 2024; Wu and You, 2023). Thus, the transition from crystal deposition to acute inflammation can be conceptualized as a “two-hit” process: First hit: MSU crystal deposition on cartilage surfaces activates synovial fibroblasts to release CCL2/MCP-1, recruiting monocytes and establishing subclinical inflammation. Second hit: Stimuli such as trauma or hypothermia cause crystal shedding. LL-37 released from neutrophil extracellular traps (NETs) binds crystals, forming pro-inflammatory complexes that trigger an IL-1β storm.

#### 2.1.7 Chronic gout and organ damage

Persistent MSU crystal deposition leads to tophi formation, joint destruction, and chronic kidney disease. Joint damage: MSU crystals within tophi chronically activate macrophages, polarizing them toward an M1 phenotype. These macrophages secrete MMP-13 and ADAMTS-5, degrading type II collagen. Concurrently, TGF-β1-mediated fibrous encapsulation drives joint structural remodeling and cartilage degradation (Towiwat et al., 2019). Renal damage: following endocytosis by renal tubular epithelial cells, MSU crystals activate the TLR9/MyD88 pathway. This promotes NADPH oxidase-derived ROS, which activates the TGF-β/Smad3 signaling pathway, ultimately leading to renal interstitial fibrosis (Wang H. et al., 2018; Luna et al., 2025; Spiga et al., 2017).

### 2.2 Pathogenesis and pathophysiology of hyperuricemia

**FIGURE 2:**
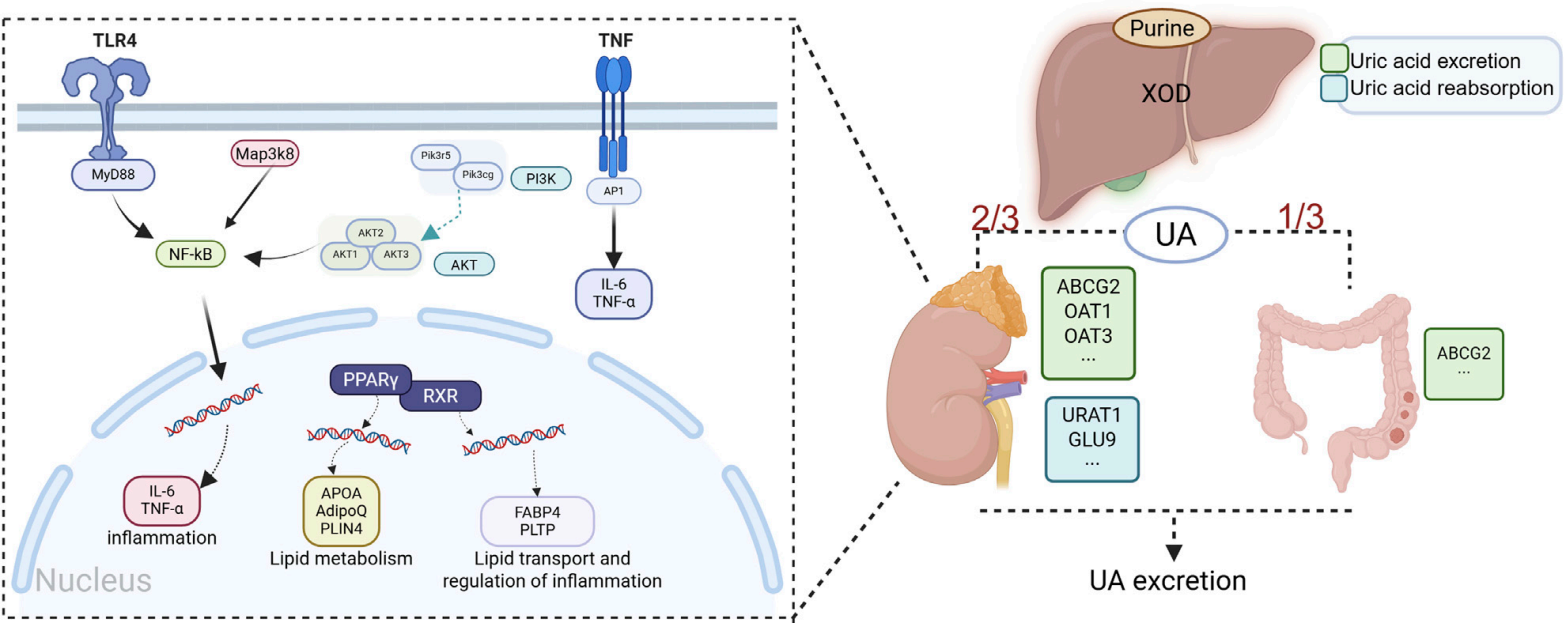
Uric acid metabolism and the metabolic–inflammatory axis in hyperuricemia. Note: UA production is catalyzed by XOD, whose activity is modulated by genetic and metabolic factors. UA excretion involves dual regulatory mechanisms: renal homeostasis maintained by URAT1/GLUT9-mediated reabsorption and OAT1-mediated secretion, coupled with intestinal ABCG2-dependent efflux. Metabolic syndrome components dysregulate the ApoA/AdipoQ/FABP4 axis and PPARγ–RXR signaling, promoting hyperuricemia via insulin resistance-induced endogenous purine synthesis and downregulation of OAT1/ABCG2, thereby reducing UA excretion. This initiates a vicious inflammatory cycle: TLR4 recognition of DAMPs activates MyD88-dependent MAP3K8/AKT signaling, inducing NF-κB nuclear translocation and upregulating IL-6/TNF-α; TNF-α further amplifies inflammation via PI3K–AP1 signaling. These cytokines directly activate the renal NLRP3 inflammasome while indirectly reducing UA excretion (through URAT1 downregulation) and promoting crystal deposition via mitochondrial oxidative stress. Impaired intestinal ABCG2 function exacerbates UA retention, reinforcing a pathogenic metabolic–inflammatory axis (created in BioRender. https://BioRender.com/mpuv1n5).

Hyperuricemia is the precursor state to gout, but its pathophysiological impact extends far beyond the joints, involving multiple systems, including cardiovascular diseases, chronic kidney disease, and metabolic disorders (Wang Q. et al., 2018; Luna et al., 2025; Spiga et al., 2017). The pathogenesis of hyperuricemia is primarily delineated into two pathophysiological mechanisms: hyperproduction of uric acid and defective renal excretion. Complementary contributing factors encompass genetic predisposition, dysregulated inflammatory pathways, and metabolic comorbidities, which collectively modulate disease expression and progression (Dumusc and So, 2024). Uric acid metabolism and the metabolic–inflammatory axis in hyperuricemia are shown in Figure 2.

#### 2.2.1 Uric acid overproduction

Abnormal purine metabolism is the primary cause of excessive uric acid production. Uric acid is the final metabolite of purines in humans. Normal adults produce approximately 700 mg of uric acid daily (Jalal et al., 2013). Endogenous purines originate from nucleic acid breakdown of damaged/apoptotic cells, along with adenine and guanine degradation, whereas exogenous purines are primarily derived from dietary sources, including fructose-rich beverages and purine-rich foods (e.g., meat and seafood) (Chaudhary et al., 2013; Weaver, 2019). These exogenous substrates undergo UA biosynthesis in the liver, intestinal, and vascular endothelial compartments. The terminal step of human purine metabolism involves XO-catalyzed oxidation of hypoxanthine to xanthine, followed by further oxidation to uric acid. Circulating UA is distributed via the bloodstream to excretory organs (kidneys and intestines) for elimination (Hille et al., 2014; Wen et al., 2024). As the rate-limiting enzyme in UA synthesis, XO activity is modulated by genetic factors (e.g., HPRT1 mutations) and dietary purine intake. Pharmacological inhibition of XO (e.g., allopurinol) demonstrates marked hypouricemic effects, underscoring the clinical significance of this pathway (Yang et al., 2024). Additionally, accelerated ATP degradation under pathological conditions (e.g., intense exercise and ischemia–reperfusion injury) promotes hypoxanthine accumulation, creating a secondary drive for UA overproduction (Uskudar Cansu et al., 2019).

#### 2.2.2 Impaired renal uric acid excretion

Renal handling of uric acid plays a pivotal role in maintaining systemic urate homeostasis. Approximately 90% of filtered uric acid undergoes reabsorption, while ∼10% is excreted in urine, with glomerular filtration occurring via passive diffusion across capillary endothelial pores (Wen et al., 2024). Renal urate transport involves three sequential processes: glomerular filtration, proximal tubule reabsorption, and active secretion. Dysregulation of urate transporters in the proximal convoluted tubule constitutes the principal determinant of net urinary excretion (Nakayama et al., 2023; Eckenstaler and Benndorf, 2021). In particular, URAT1 (SLC22A12) and OAT10 (SLC22A13) serve as the primary mediators of apical reabsorption, whereas basolateral secretion involves ATP-binding cassette subfamily G member 2 (ABCG2) and sodium-dependent phosphate transporter 1 (NPT1/SLC17A1). Furthermore, metabolic comorbidities such as insulin resistance indirectly enhance urate reabsorption through NHE3-mediated (sodium–hydrogen exchanger 3) tubular alkalinization, creating a mechanistic link between metabolic syndrome and hyperuricemia (Gong et al., 2020; Abrahim, 2021).

#### 2.2.3 Regulatory role of intestinal excretion

Quantitative contribution: Using C14 tracer studies, Sorensen (1965) demonstrated that a significant portion of total body uric acid turnover (∼1/3) is excreted via the intestine (extrarenal elimination pathway) compared to ∼2/3 excreted renally (Du et al., 2024). ABCG2 function: ABCG2, which is highly expressed on the apical membrane of intestinal epithelial cells, plays a critical role in intestinal urate secretion. Its dysfunction can reduce intestinal urate excretion by >50%, significantly elevating serum urate levels. Gut microbiota: Animal models suggest that gut microbiota (e.g., Bifidobacterium) can modulate host urate metabolism by degrading urate precursors, highlighting the potential role of the gut–kidney axis in hyperuricemia (Cabau et al., 2023).

#### 2.2.4 Mechanisms of renal and intestinal urate reabsorption and excretion

Renal and intestinal handling of urate relies on specific transporters to maintain systemic urate levels. These are categorized as follows. Reabsorptive transporters: e.g., glucose transporter 9 (GLUT9/SLC2A9) and urate transporter 1 (URAT1/SLC22A12). Secretory transporters: e.g., organic anion transporters OAT1 and OAT3 (SLC22A6 and SLC22A8), ATP-binding cassette transporter G2 (ABCG2), sodium-dependent phosphate transporter 1 and 4 (NPT1/SLC17A1 and NPT4/SLC17A3), and multidrug resistance protein 4 (MRP4/ABCC4). Renal localization (proximal tubule): reabsorption: URAT1 (apical membrane), GLUT9 (apical and basolateral membranes), and OAT4/10 (mainly apical). Secretion: ABCG2, OAT1/3 (basolateral), MRP4, and NPT1/4 (apical) ensure urate secretion from blood into the tubular lumen (Enomoto et al., 2002; Hu Q. et al., 2024; Yu et al., 2023; Bahn et al., 2008). Intestinal localization: GLUT9 is expressed on the apical and basolateral membranes of murine intestinal epithelial cells. ABCG2 (apical membrane of enterocytes) and NPT1 (apical membrane of small intestinal cells) are also significant intestinal urate transporters (Chen et al., 2019).

#### 2.2.5 Inflammation, oxidative stress, and others

Asymptomatic hyperuricemia exhibits pro-inflammatory and pro-oxidative properties that contribute to multisystem pathology. Elevated SUA activates endothelial NF-κB signaling, upregulating monocyte chemoattractant protein-1 (MCP-1) and intercellular adhesion molecule-1 (ICAM-1) expressions to accelerate atherosclerosis progression (Wang H. et al., 2018; Spiga et al., 2017). Furthermore, microcrystalline urate deposition activates the NLRP3 inflammasome in renal and vascular tissues, exacerbating kidney disease and cardiovascular complications through IL-1β-mediated inflammation (Cabau et al., 2023; Wang Q. et al., 2018).

This metabolic disturbance demonstrates bidirectional interactions with components of metabolic syndrome. Adipose tissue-derived free fatty acids (FFAs) impair renal urate excretion via URAT1 upregulation, while hyperuricemia reciprocally induces insulin resistance and endothelial dysfunction through AMPK inhibition and oxidative stress (Xu et al., 2015). This vicious cycle creates a “metabolic hyperuricemia” phenotype, characterized by stimulated hepatic lipogenesis and dysregulated adipokine levels. Pharmacological contributors include thiazide diuretics (which inhibit renal urate secretion), cyclosporine (which reduces glomerular filtration), and pyrazinamide (which increases purine synthesis), all of which can precipitate secondary hyperuricemia (Zhang K. et al., 2019). Such drug-induced elevations compound the effects of systemic comorbidities, including hypertension, insulin resistance, and atherosclerosis, collectively impairing urate homeostasis through both enhanced production and reduced excretion.

Thus, the pathogenesis of gout is fundamentally driven by hyperuricemia, which serves as both a precursor and a central pathological hub. This condition involves intricate regulation across genetic, epigenetic, metabolic, and immune dimensions, creating a multifactorial disease network. As an independent pathological entity, hyperuricemia extends beyond gout pathogenesis to contribute to systemic comorbidities, including atherosclerosis, chronic kidney disease, and metabolic syndrome. This broad pathophysiological impact underscores the necessity of holistic therapeutic strategies targeting both urate-lowering and anti-inflammatory mechanisms.

## 3 Diagnosis and treatment of gout and hyperuricemia

### 3.1 Diagnosis of gout

The diagnosis of gout requires integration of clinical manifestations, laboratory findings, and imaging evidence. According to the classification criteria jointly developed by the American College of Rheumatology (ACR) and the European Alliance of Associations for Rheumatology (EULAR), the identification of MSU crystals in synovial fluid is the diagnostic “gold standard” (Liang et al., 2024; Morlock et al., 2025). For patients where synovial fluid analysis is unfeasible, imaging techniques such as ultrasound and dual-energy computed tomography (DECT) can detect urate deposition, with sensitivities ranging from 15% to 40%. These techniques hold particular value for the early identification of asymptomatic hyperuricemia (Morlock et al., 2025; Dalbeth et al., 2021). The recently developed MSU Crystal Deposition Quantification (MSKF) scoring system, which quantifies urate burden based on ultrasound features (e.g., double-contour sign and tophi), has been validated for distinguishing asymptomatic hyperuricemia from gout. Laboratory diagnosis typically involves SUA levels ≥6.8 mg/dL (404 μmol/L), which represents a critical biochemical prerequisite for gout development (Zhu et al., 2025). However, approximately 50% of patients with chronic hyperuricemia do not progress to clinical gout within 15 years (Singh et al., 2025). Therefore, guidelines emphasize the need to exclude other crystal-induced arthropathies (e.g., pseudogout) and consider the diagnosis in conjunction with typical symptoms such as joint swelling, redness, heat, and pain (Panlu et al., 2024; Morlock et al., 2025).

### 3.2 Pharmacological treatment of gout

#### 3.2.1 Acute gout flare management

Pharmacotherapy for gout addresses both acute inflammation/pain during flares and long-term urate lowering in the chronic phase. First-line agents for acute flares include the following: NSAIDs: reduce inflammation by inhibiting COX. Potential gastrointestinal and cardiovascular risks must be considered (Bartsch et al., 2025; Yao et al., 2024). Colchicine: exerts anti-inflammatory effects by inhibiting microtubule polymerization and neutrophil chemotaxis (Quintana et al., 2023). A low-dose regimen (e.g., 1.2 mg initially, followed by 0.6 mg after 1 h) is recommended to minimize side effects such as diarrhea. Long-term low-dose colchicine can also be used for flare prophylaxis. Glucocorticoids: effective for acute inflammation, often used orally or via intra-articular injection, especially when NSAIDs or colchicine are contraindicated (Bartsch et al., 2025; Forster, 2024).

#### 3.2.2 ULT for chronic management

Chronic management centers on ULT, using agents from several classes. XO inhibitors (XOIs). Allopurinol: the most commonly used first-line agent, which inhibits uric acid production. Careful monitoring is required due to the risk of hypersensitivity reactions (e.g., Stevens–Johnson syndrome) (Dillman et al., 2024). Febuxostat: a selective XOI suitable for patients intolerant to allopurinol, although associated with an increased risk of cardiovascular events (Quintana et al., 2023). Uricosuric agents. Benzbromarone: promotes renal uric acid excretion by inhibiting tubular reabsorption (primarily via URAT1) (Pino-Zambrano et al., 2024). It is contraindicated in patients with renal stones or significant renal impairment. Uricase agents. Pegloticase: indicated for refractory gout, catalyzes the oxidation of uric acid to highly soluble allantoin, rapidly lowering SUA. Monitoring is required for infusion reactions and the potential development of anti-drug antibodies.

#### 3.2.3 Natural products and plant-derived therapeutics

Research on natural compounds shows promise, although clinical application often faces challenges. Theaflavins: exhibit anti-gout potential by inhibiting inflammatory pathways (e.g., NLRP3 inflammasome) and modulating uric acid metabolism. Limited bioavailability remains a hurdle (Liu G. et al., 2025; Yan et al., 2025). Dioscorea septemloba: network pharmacology analysis suggests that its active components may regulate inflammation and urate metabolism through multiple targets (Liu W. et al., 2025). Taxifolin (dihydroquercetin): shows promise in reducing inflammatory responses and acute flare frequency, particularly when combined with dietary interventions (Piao et al., 2022). In addition, various other natural products have demonstrated efficacy in treating gout and will not be individually listed here.

#### 3.2.4 Exploratory non-pharmacological approaches

Emerging non-pharmacological strategies are under investigation. Probiotics: modulation of the gut microbiota (e.g., using Limosilactobacillus reuteri) has been associated with reduced SUA levels and decreased gout flare frequency (Rodriguez et al., 2023). Transcutaneous auricular vagus nerve stimulation: this technique, which aims to suppress systemic inflammation, may serve as a potential non-pharmacological approach for managing chronic inflammation in the future (Shin et al., 2024). In addition, non-pharmacological interventions for gout, including dietary modifications, weight management, and physical therapies, demonstrate multi-target efficacy by reducing serum uric acid levels, modulating inflammatory pathways, and improving metabolic parameters, thereby decreasing flare frequency, preventing complications, and complementing pharmacotherapy in mild-to-moderate cases.

### 3.3 Diagnosis of hyperuricemia

Hyperuricemia is defined as SUA levels exceeding 7.0 mg/dL (416 μmol/L) in male individuals and 5.6 mg/dL (333 μmol/L) in female individuals (Wu F. et al., 2024; Tuono et al., 2024). Diagnostic evaluation requires integration of SUA measurements with metabolic syndrome assessment (e.g., obesity and insulin resistance) (Ji et al., 2025). Notably, ultrasonography or DECT reveals urate crystal deposition in 15%–40% of asymptomatic hyperuricemic patients, indicating subclinical gout risk (Xiang et al., 2024; He et al., 2024). However, the demarcation between asymptomatic hyperuricemia and gout remains clinically ambiguous as only approximately 50% of patients with prolonged hyperuricemia develop gout within 15 years (Xiang et al., 2024; Chang et al., 2025). This underscores the necessity of imaging modalities (e.g., DECT) for detecting urate deposition to differentiate subclinical gout (Chang et al., 2025). The definitive diagnostic criterion for gout relies on the identification of MSU crystals in synovial fluid or tissues, with elevated SUA serving solely as a supplementary indicator.

### 3.4 Pharmacological treatment of hyperuricemia

#### 3.4.1 Conventional agents and mechanisms of action

Pharmacotherapy for hyperuricemia involves multiple mechanisms and drug classes, primarily targeting urate production inhibition, uricosuric effects, and metabolic pathway modulation (Du et al., 2024). XOIs represent the cornerstone of conventional treatment. Allopurinol, a classic XOI, reduces urate synthesis by inhibiting hepatic xanthine oxidoreductase (XOR) (Wu D. et al., 2024). Clinical studies demonstrate that allopurinol combined with febuxostat improves the glomerular filtration rate in non-dialysis CKD patients (Nguyen et al., 2024). Febuxostat, a novel selective XOR inhibitor, exhibits superior efficacy to non-pharmacological interventions in asymptomatic hyperuricemia trials, particularly in ameliorating arterial stiffness assessed using cardio-ankle vascular index (CAVI) (Kawachi et al., 2025). Uricosuric agents, exemplified by benzbromarone, enhance urinary urate excretion via regulation of renal transporters (e.g., OAT1), although hepatotoxicity limits chronic use (Sun et al., 2025).

#### 3.4.2 Natural products and botanical-derived therapies

Natural products are emerging as alternatives to conventional medications. Traditional Chinese medicine formulations, such as Simiao Pills, demonstrate multi-component synergy (phenolic acids, terpenoids, and alkaloids) in mitigating hyperuricemia and renal injury, mediated through anti-inflammatory effects and renal transporter modulation (Li et al., 2022; Zeng et al., 2024). Atractylodes macrocephala exhibits anti-hyperuricemic and anti-inflammatory properties in animal models, reducing IL-1β and TNF-α levels while activating AMPK/SIRT1 signaling and suppressing NF-κB-mediated macrophage polarization toward pro-inflammatory phenotypes (Qian et al., 2023). Polyphenolic compounds, including hazel leaf polyphenols, exert dual inhibitory effects on XOR activity and OAT1/hypoxanthine-guanine phosphoribosyltransferase (HPRT) gene expression, with molecular docking studies predicting PI3K–AKT pathway involvement (Mehmood et al., 2022; Wang R. et al., 2024). Naringenin demonstrates dose-dependent urate-lowering and anti-inflammatory renal effects by inhibiting XOR and modulating inflammatory pathways (Yang Y. et al., 2023). Additionally, isobavachin (derived from Pittosporum species) acts as a human urate transporter 1 (hURAT1) inhibitor, blocking urate reabsorption to reduce SUA levels, akin to the mechanism of tranilast (Luo et al., 2023).

#### 3.4.3 Exploratory non-pharmacological approaches

Non-pharmacological approaches serve as essential adjuncts for hyperuricemia management, particularly in asymptomatic individuals (Paul et al., 2017; Valsaraj et al., 2020). Key dietary modifications include the following. Restriction: limiting alcohol (especially beer/spirits), sugar-sweetened beverages, heavy meat/seafood consumption, and excessive purine intake (reducing SUA by ∼1 mg/dL) (Mitnala et al., 2016). Incorporation: emphasizing cherries, coffee, low-fat dairy, and moderate consumption of protein-rich vegetables (nuts, legumes, and spinach) due to their low urate bioavailability and beneficial fiber (Levy and Cheetham, 2015; Zhang et al., 2012; Park et al., 2016). Supplementation: Vitamin C demonstrates SUA-lowering potential (Peng et al., 2024). Weight management remains fundamental (Du et al., 2024). Although some guidelines support treating asymptomatic hyperuricemia to mitigate hypertension, CKD, and CAD risks, treatment initiation requires individual risk stratification pending further validation of cardiovascular/renal benefits (Waheed et al., 2021; Chales, 2019).

### 3.5 Limitations of conventional therapeutic paradigms for gout and hyperuricemia

Current clinical management of gout and hyperuricemia faces multifaceted challenges, with therapeutic bottlenecks stemming from the following critical conflicts.

#### 3.5.1 Safety–efficacy trade-offs

Although conventional urate-lowering agents (e.g. XOI allopurinol and URAT1 inhibitor benzbromarone) effectively reduce SUA levels, their clinical utility is constrained by significant organ toxicity risks (Wu F. et al., 2024). Allopurinol-induced hypersensitivity syndrome, characterized by exfoliative dermatitis and liver failure, arises from purine metabolism inhibition (Alotaibi et al., 2024). Benzbromarone exacerbates renal burden through enhanced uricosuria, potentially precipitating tubulointerstitial fibrosis in CKD patients (Kang et al., 2024). These safety concerns necessitate dose limitations, compromising sustained target inhibition efficacy.

#### 3.5.2 Inadequate precision in metabolic regulation

Emerging evidence highlights the multi-organ (hepatic–renal–intestinal) coordination of urate metabolism, yet existing pharmacotherapies predominantly target single nodes (e.g., XO or URAT1). This limitation is particularly pronounced in obesity-associated gout, where XOIs fail to modulate aberrant glycolytic pathways (Elsayed and Elsaid, 2022). Altered activity of glycolytic enzyme GAPDH may promote urate production via perturbation of purine intermediates (e.g., 5-phosphoribosyl-1-pyrophosphate) (Jezewski et al., 2022). Obesity-related insulin resistance activates a glycolysis–purine metabolic axis, driving precursor accumulation that remains unaddressed by current therapies (Panlu et al., 2024). Furthermore, elevated XO activity correlates with adipose tissue dysfunction, potentially exacerbating insulin resistance through ROS-NF-κB signaling (Ali et al., 2024). This “single-target” intervention model demonstrates limited efficacy in obesity-related gout, where metabolic memory effects precipitate rapid urate dysregulation post-treatment cessation. Animal studies demonstrate compensatory upregulation of hepatic purine metabolic enzymes (e.g., PRPP synthetase) following XOI discontinuation, while clinical observations reveal more pronounced SUA rebound in obese gout patients than in non-obese counterparts—a phenomenon potentially mediated by free fatty acid-induced hepatic XO activation from adipose tissue (Cheng L. et al., 2025).

#### 3.5.3 Therapeutic adherence challenges

Chronic conditions (e.g., gout, diabetes, and psychiatric disorders) require prolonged pharmacotherapy (e.g., febuxostat or antipsychotics), yet patient adherence wanes with complex regimens (e.g., twice-daily dosing) (Bartsch et al., 2025; Cai et al., 2024). Long-acting injectable (LAI) formulations reduce the dosing frequency but exhibit variable adherence in real-world practice, with some patients discontinuing therapy due to perceived complexity (Lnu et al., 2024). Concomitant monitoring requirements (SUA levels and liver/renal function) impose additional burdens: frequent phlebotomy provokes patient resistance, while toxicity surveillance (ALT, AST, and creatinine) demands individualized schedules that increase healthcare encounters (Kaur et al., 2024; Wu D. et al., 2024; Park et al., 2024; Boruah et al., 2024). Special populations (e.g., post-renal transplant and CKD patients) require intensified monitoring, further exacerbating compliance challenges (Boruah et al., 2024).

### 3.6 Innovative potential and clinical translation of DDS

Current advancements in machine learning, bioinformatics, and multi-omics technologies have enabled significant progress in targeted precision interventions for cancer (Shao et al., 2025; Yan et al., 2024). However, precision research on immunometabolic diseases such as gout and hyperuricemia still predominantly relies on DDS. Addressing the limitations of conventional therapies, DDS offers a three-dimensional therapeutic paradigm through interdisciplinary innovations in material science and pharmacokinetics, particularly for gout and hyperuricemia management (Peng et al., 2023).

#### 3.6.1 Organ-specific targeted delivery

Surface-modified nanocarriers (e.g., nanoparticles and liposomes) enable precise drug enrichment in renal proximal tubules or hepatocytes (Peng et al., 2023). Receptor-mediated targeting: mannose receptor-targeted nanocarriers (e.g., liposomes and polymeric nanoparticles) decorated with URAT1-specific ligands demonstrate enhanced tubular accumulation, minimizing gastrointestinal adverse effects associated with systemic drug exposure (Faustino et al., 2023). Trans-physiological barrier delivery: nanoscale carriers overcome limitations of conventional agents in penetrating barriers (e.g., blood–brain barrier and synovial membrane) through size-dependent diffusion and active targeting moieties (e.g., hyaluronic acid-modified silk fibroin nanoparticles), prolonging drug retention at inflammatory sites (Cheng Z. et al., 2025; Garhwal et al., 2024). Toxicity reduction: targeted delivery mitigates hepatotoxicity and hypersensitivity risks inherent to non-selective agents (e.g., allopurinol) while improving therapeutic indices (Ezike et al., 2023).

#### 3.6.2 Spatiotemporally controlled sustained-release platforms

Microsphere/liposome systems: PLGA- or lipid-encapsulated uricase formulations achieve weeks-to-months sustained release, reducing injection frequency and improving adherence in chronic regimens (Ezike et al., 2023). Transdermal systems: novel platforms (e.g., silk fibroin/hyaluronic acid microneedles and chitosan patches) bypass gastrointestinal metabolism, maintaining cutaneous drug concentrations for >7 days—particularly advantageous for obese patients or those with venous access limitations (Harwansh et al., 2023). Environment-responsive release: pH-sensitive nanocarriers (e.g., FeO/chitosan/TiO_2_ composites) trigger drug release in acidic microenvironments (e.g., inflammation sites and crystal deposits), enhancing local efficacy (Mohamadi Bian and Moghadam, 2025; Liu et al., 2024).

#### 3.6.3 Multimodal synergistic therapeutics

DDS enables “urate-lowering + anti-inflammatory” combinatorial strategies through co-delivery systems. Dual-action nanocarriers: liposomal or polymeric nanoparticles co-encapsulating IL-1β inhibitors and XOIs (e.g., febuxostat) achieve sequential neutralization of inflammatory cytokines and sustained XO inhibition, preventing crystal formation (Liu Y. et al., 2023; Wang et al., 2025). Immunomodulatory integration: nano-formulated PD-1 blockade therapies modulate macrophage polarization and suppress NLRP3 inflammasome activation, mitigating chronic inflammatory damage in gouty arthritis (Liu Y. et al., 2023).

Gout and hyperuricemia management requires integrated approaches addressing metabolic dysregulation and organ-specific damage. Conventional therapies fall short in high-risk populations (e.g., obesity and renal impairment) due to toxicity and adherence limitations. DDS innovations—through pharmacokinetic optimization, precision targeting, and combinatorial strategies—represent transformative solutions to overcome these barriers, offering safer, more effective therapeutic options for complex clinical scenarios.

## 4 Application of DDS in gout and hyperuricemia

### 4.1 Introduction to DDSs and its classification in gout/hyperuricemia management

DDSs represent engineered platforms designed to optimize drug pharmacokinetics and biodistribution through advanced carrier technologies (e.g., nanoparticles, colloidal systems, and polymers). Their primary objectives include enhancing bioavailability, improving targeting efficiency, and enabling controlled release profiles to maximize therapeutic efficacy while minimizing off-target toxicity (Garhwal et al., 2024). For instance, nanomedicine-based DDSs (NDDSs) leverage nanoscale architectures to achieve superior target specificity compared to conventional formulations, which often suffer from poor bioavailability and non-selective distribution (Losada-Barreiro et al., 2024). Key DDS design parameters encompass physicochemical properties (e.g., particle size, surface charge, and stability) that directly influence delivery efficiency and safety profiles (Ju and Cho, 2023).

DDS applications span diverse disease domains and administration routes. In oncology, extracellular vesicle-based systems enhance drug penetration into tumor microenvironments, mitigating chemotherapy resistance (Zheng et al., 2024). Ophthalmic lipid-based formulations (e.g., liposomes) improve intraocular drug retention through sustained-release mechanisms (Batur et al., 2024). Pulmonary nanocarriers optimize aerosolized drug deposition in respiratory diseases, while chitosan-based systems enable chronic disease management via mucoadhesive drug delivery (Jeong et al., 2025; Manohar et al., 2025). β-Glucan-functionalized carriers demonstrate efficacy in inflammatory and immune-mediated disorders through biocompatibility-driven targeting (Wu et al., 2023). Collectively, DDS embodies an engineering-driven paradigm focused on precision medicine through tailored carrier design (e.g., polymeric micelles and biomimetic nanodevices).

Recent advances in DDS research for gout and hyperuricemia reflect a paradigm shift toward precision therapy (Karantas et al., 2024). Current classification frameworks categorize DDS into three principal classes: lipid-based, polymer-based, and other systems. These platforms enhance therapeutic performance through targeted delivery, controlled release kinetics, and barrier penetration capabilities. Lipid-based DDSs (LBDDSs) utilize phospholipid bilayer architectures [e.g., liposomes, ethosomes, and solid lipid nanoparticles (SLNs)] to achieve localized drug enrichment in joint cavities or inflammatory foci, with reduced immunogenicity due to biomimetic membrane properties (Seo et al., 2023). Polymer-based DDSs use synthetic (e.g., PLGA) or natural polymers (e.g., chitosan) to fabricate nanocapsules, hydrogels, or microneedle arrays, enabling prolonged drug release profiles particularly advantageous for chronic hyperuricemia management (Behnke et al., 2024). Hybrid systems (e.g., inorganic nanoparticles and EV–mimetic carriers) integrate multifunctional materials or biological motifs to overcome limitations of conventional carriers (Ng et al., 2022).

### 4.2 LBDDSs for gout and hyperuricemia

LBDDSs represent a class of platforms utilizing lipid materials to enhance drug bioavailability, stability, and targeting efficiency (Savla et al., 2017). Key categories include liposomal systems, SLNs, ethosomal carriers, and self-emulsifying drug delivery systems. These systems encapsulate both hydrophilic and lipophilic drug molecules, improving solubility, absorption, and delivery efficacy across biological barriers. LBDDSs have demonstrated broad applicability in oral, transdermal, topical, and systemic administration for diseases including cancer, autoimmune disorders, and dermatological conditions (Liu X. et al., 2025).

#### 4.2.1 Liposomal systems and functional derivatives

Liposomal systems encompass conventional vesicles and functionalized derivatives (e.g., biomimetic nanoliposomes and elastic liposomes). Composed of phospholipid bilayers, liposomes exhibit biodegradability, nontoxicity, and dual drug-loading capacity for hydrophilic and lipophilic agents (Danaei et al., 2018; Breitsamer and Winter 2019). Their established manufacturing processes enable sustained drug release, active targeting, and combinatorial therapy delivery (Salunkhe et al., 2021). In gout research, liposomal encapsulation of eremantholide C and goyazensolide reduced systemic toxicity while preserving anti-hyperuricemic activity, as evidenced by stabilized SUA reduction and biocompatibility in preclinical models (Ma et al., 2025). For hyperuricemia management, PEGylated liposomal uricase demonstrated enhanced oral bioavailability by protecting enzymes from gastrointestinal degradation and facilitating intestinal epithelial transcytosis (Nishida et al., 1984). Notably, PEGylation prevented immune complex formation and anti-uricase antibody induction in avian models, underscoring its translational potential. Liposomal encapsulation of eremantholide C and goyazensolide in a sesquiterpene formulation mitigates toxicity while maintaining anti-hyperuricemic efficacy through enhanced encapsulation and sustained release, with preclinical evaluation confirming serum uric acid stabilization, excellent biocompatibility, and therapeutic potential for concurrent hyperuricemia/gout management, warranting translational investigation (Cunha Matosinhos et al., 2024).

Biomimetic nanoliposomes represent a cutting-edge subset of LBDDSs. For instance, USM [H]L vesicles integrate encapsulated enzymes to degrade uric acid and hydrogen peroxide, generate photothermal effects for inflammation disruption, repolarize M1 macrophages to M2 phenotypes, and reprogram metabolic/immunological pathways for gout resolution (Chen et al., 2023). Similarly, biomimetic nanovesicles encapsulating uricase achieved 533% and 331% bioavailability improvements compared to native enzymes, with UHLNX formulations demonstrating rapid SUA normalization superior to free uricase (Yang et al., 2020).

Elastic liposomes (ultradeformable liposomes or transfersomes) exhibit exceptional deformability, enabling transdermal penetration through skin barriers (Kumar and Utreja, 2020). Their enhanced permeability profiles reduce systemic side effects in applications such as antihypertensive and topical antibiotic delivery (Yang M. et al., 2019). In gout models, elastic liposomes improved colchicine efficacy by increasing stratum corneum permeation, sustaining drug release to reduce MSU crystal-induced exudate volume and leukocyte infiltration, and inhibiting collagen deposition/inflammatory cell accumulation (Singh et al., 2009).

#### 4.2.2 SLNs and nanostructured lipid carriers

SLNs represent a lipid-based delivery platform characterized by a solid lipid matrix that enables sustained drug release and enhanced chemical stability (Duan et al., 2020). SLNs offer biodegradability, nontoxicity, and superior physical stability compared to liposomes, with simplified manufacturing processes that minimize drug leakage (Duan et al., 2020; Silva et al., 2022). These carriers improve the solubility of poorly water-soluble drugs and accommodate multiple therapeutic agents, making them versatile for oral, transdermal, and topical applications (Mahmoudian et al., 2021). Studies demonstrate that SLNs significantly enhance the solubility and oral bioavailability of [6]-shogaol, a ginger-derived compound. In hyperuricemic/gouty arthritis rat models, SLN-encapsulated [6]-shogaol exhibited superior efficacy compared to the free drug, marked by greater reductions in serum uric acid, IL-1β, and TNF-α levels. Mechanistically, [6]-shogaol reduces urate production through XOD inhibition, while SLN encapsulation provides multi-organ protective effects by mitigating drug degradation and improving biodistribution. These findings position SLNs as promising vehicles for enhancing the anti-gout and urate-lowering properties of hydrophobic compounds (Wang H. et al., 2018).

Nanostructured lipid carriers (NLCs), second-generation lipid systems, incorporate mixed solid–liquid lipid matrices to reduce crystallinity and improve drug loading capacity compared to SLNs (Mahmoudian et al., 2021). This structural optimization minimizes drug expulsion during storage and enhances delivery efficiency (Ashkar et al., 2022). In gout therapy, NLCs demonstrate biocompatibility across administration routes, with controlled-release formulations improving solubility and bioavailability of XOIs (e.g., febuxostat). For instance, NLC-encapsulated febuxostat achieves sustained urate-lowering effects while reducing gastrointestinal side effects associated with oral delivery (Sharma et al., 2022). The hybrid lipid composition of NLCs enables tailored release kinetics, making them advantageous for chronic disease management requiring long-term pharmacotherapy.

#### 4.2.3 Ethosomal systems for transdermal management

Ethosomes represent ethanol-based vesicular carriers characterized by enhanced skin permeation properties, leveraging high ethanol concentrations to disrupt stratum corneum resistance and facilitate drug delivery to epidermal and dermal layers (Kumar and Utreja, 2020). These systems demonstrate superiority in topical anti-inflammatory, antimicrobial, and antihypertensive therapies through increased cutaneous drug deposition (Kumar and Utreja, 2020). Ethosomes’ biodegradability and formulation simplicity enable integration with other carriers for synergistic performance optimization. For instance, a soluble nanoneedle system co-encapsulating colchicine and iguratimod in ethosomes, combined with borneol-modified colchicine ethosomes, achieves dual-mechanism gout treatment. This transdermal platform integrates nanoneedle-mediated osteoclast inhibition/cartilage preservation with borneol-enhanced permeation to suppress MSU crystal-induced inflammation. By stabilizing drug bioavailability while reducing neutrophil activation, pro-inflammatory cytokine secretion, and osteoclast activity, the formulation minimizes gastrointestinal toxicity, offering superior efficacy in managing gout-related pain, joint destruction, and systemic complications compared to conventional oral therapies (Li Z. et al., 2024; Zhang S. et al., 2019).

Ethosomal gels and cataplasms represent semi-solid formulations that enhance topical drug delivery through matrix-controlled release kinetics and prolonged skin retention. As novel transdermal platforms, these systems enable multi-target gout therapy by combining alpha-phellandrene with colchicine (Valsalan Soba et al., 2021). Alpha-phellandrene-loaded cellulose gels block inflammatory mediator synthesis via COX-II and LOX-5 inhibition, while colchicine cataplasms inhibit MSU crystal-induced cytokine release and neutrophil recruitment through transdermal pathways (Valsalan Soba et al., 2021). This dual-component system synergistically reduces inflammation through enzyme inhibition and immunomodulation while significantly mitigating gastrointestinal toxicity and systemic drug exposure, providing a bone-protective strategy with enhanced penetration efficacy for gout-related pain and joint damage.

#### 4.2.4 Self-emulsifying drug delivery systems

Self-emulsifying drug delivery systems (SEDDSs) represent lipid-based formulations that spontaneously form emulsions or microemulsions in gastrointestinal environments, including self-nanoemulsifying DDSs and self-microemulsifying DDSs (SMEDDSs), significantly enhancing the oral bioavailability of lipophilic drugs (Buya et al., 2021). These systems simplify formulation development for poorly soluble compounds by improving intestinal solubility and permeability through emulsion-mediated transport. Lipid composition optimization enables drug stabilization, targeted delivery, and controlled release while minimizing *ex vivo* degradation (Haddadzadegan et al., 2022; Jampilek and Kralova, 2022). In gout therapy, SEDDSs/SMEDDSs demonstrate significant potential for optimizing pharmacokinetics and enhancing therapeutic indices. Febuxostat-loaded SEDDSs/self-nanoemulsifying lipid transporters (SNELTs) improve XOD inhibitory efficacy by augmenting drug solubility and intestinal absorption, achieving sustained urate-lowering effects with 146.4% relative bioavailability and reduced gastrointestinal toxicity in gouty arthritis models. These formulations optimize pharmacokinetic parameters (elevated Cmax and prolonged AUC) while enabling less frequent dosing, underscoring their clinical utility for managing hyperuricemia-driven inflammation (Rangaraj et al., 2019; Al-Amodi et al., 2020).

In addition, complementary mechanisms provide support for the efficacy of SEDDSs/SMEDDSs in hyperuricemia, as detailed below. Liquiritin–SEDDSs enhance dissolution and multi-organ protection, while morin–phospholipid SMEDDSs synergistically suppress hepatic XOD/XDH expression and restore renal function of uric acid transporters (e.g., mGLUT9 and mURAT1) (Wei et al., 2022). Similarly, 6-shogaol-SMEDDSs achieve 571.18% bioavailability enhancement, targeting XOD inhibition and reducing renal pathology via preferential liver/kidney accumulation (Yang Q. et al., 2019). Isoliquiritigenin– and licochalcone A–SMEDDS further validate this approach, demonstrating XOD suppression, lymphatic transport enhancement, and mitigation of hyperuricemia-induced organ damage (Zhang Y. et al., 2019). Collectively, these systems exemplify how SEDDSs/SMEDDSs enable multifaceted urate control through bioavailability optimization and pathophysiological targeting, offering superior efficacy-to-safety ratios compared to conventional formulations.

In Tables 1 and 2, detailed information on the application of LBDDSs in gout and hyperuricemia is provided. LBDDSs offer transformative potential for gout and hyperuricemia management by enhancing solubility, targeting efficiency, and therapeutic safety. However, critical barriers hinder their clinical translation: (1) regulatory and scalability challenges, such as standardizing sterilization/long-term stability for liposomes/SLNs, require rigorous evaluation of immune responses (e.g., PEGylated liposomal uricase) and cost-effectiveness for advanced systems (biomimetic nanoliposomes and ethosomal gels); (2) preclinical-to-clinical gaps persist in dose optimization (e.g., PK/PD modeling for febuxostat–SEDDSs) and delivery route validation (e.g., human skin studies for elastic liposomes), with limited data on patient compliance and real-world applicability; and (3) technical limitations include liposomal drug leakage, SLN/NLC matrix stability, and ethanol-induced irritation from ethosomes. Future priorities should focus on multi-center trials for safety/PK-PD profiling, biodegradable/low-cost lipid matrix development, and omics-guided biomarker discovery to bridge translational gaps, ensuring that LBDDSs achieve their precision medicine potential in gout and hyperuricemia.

**TABLE 1:** Summary of DDS treatment methods for gout.

NO	Type	Drug	Formulation	Route of administration	Anti-gout mechanism	Clinical or preclinical trials	Reference
1	Lipid-based DDS	Biomimetic Melatonin	Liposomes	Intravenous	Macrophage membrane-coated melatonin-loaded liposomes enable metabolic reprogramming of inflammatory macrophages by shifting their energy metabolism from glycolysis to oxidative phosphorylation, thereby modulating pathogenic macrophage phenotypes and ultimately attenuating acute gouty arthritis pathology.	In vivo (gouty rat arthritis model)	Ma et al. (2025)
2		Sesquiterpene	Liposomes	Oral delivery	Liposomal formulations containing sesquiterpene lactones for the treatment of chronic gout, liposomal encapsulation of eremantholide C and goyazensolide leverages high encapsulation efficiency and sustained release properties to mitigate drug toxicity while preserving antihyperuricemic activity, demonstrating consistent reduction of serum uric acid levels and favorable biocompatibility profiles.	In vivo (gouty rat arthritis model)	Cunha Matosinhos et al. (2024)
3		Neutrophil microvesicles	Liposomes	Injection	PMN-Ecto suppress gout inflammation by blocking C5a-driven IL-1β release and neutrophil influx via MerTK, while also inducing TGFβ (though insufficient alone in vivo), with their anti-inflammatory effects replicated by PS-liposomes through MerTK-dependent pathways.	In vivo (MSU crystal-induced murine peritonitis model)	Cumpelik et al. (2016)
4		Uricase, nanozyme and methotrexate	biomimetic nanosized liposome	Intravenous	USM[H]L degrades uric acid and hydrogen peroxide via encapsulated enzymes, generates photothermal effects to disrupt inflammation, repolarizes M1 macrophages to M2 phenotypes, and reprograms metabolic/immunological pathways (e.g., purine metabolism) to resolve gout.	In vivo (gouty rat arthritis model)	Chen et al. (2023)
5		Colchicine	Elastic liposomes	Transdermal delivery	Elastic liposomes enhance colchicine’s anti-gout efficacy by increasing skin penetration and deposition, sustaining drug release to reduce MSU-induced exudate volume and leukocyte infiltration, and inhibiting collagen deposition and inflammatory cell accumulation.	In vivo (gouty rat arthritis model)	Singh et al. (2009)
6		[6]-Shogaol	Solid lipid nanoparticles	Oral delivery	SLNs significantly enhanced [6]-shogaol's solubility and oral bioavailability, demonstrating superior efficacy in hyperuricemic/gouty arthritis models by markedly reducing serum uric acid, IL-1β, and TNF-α levels compared to free [6]-shogaol. Mechanistically, SLNs synergized [6]-shogaol's xanthine oxidase inhibition with augmented anti-inflammatory effects, while attenuating multi-organ damage.	In vivo (gouty rat arthritis model)	Wang Q. et al. (2018)
7		Febuxostat	Nanostructured Lipid Carriers	Transdermal delivery	Febuxostat inhibits xanthine oxidase to reduce uric acid synthesis, while NLCs enhance its solubility and bioavailability via controlled release. This dual approach ensures sustained urate-lowering efficacy and minimizes gastrointestinal side effects compared to oral delivery.	In vitro study	Sharma et al. (2022)
8		Colchicine and iguratimod	Ethosomes	Transdermal delivery	A soluble nanoneedle system co-encapsulating colchicine and iguratimod ethosomes to modulate inflammatory cytokines, suppress osteoclast activity, and synergistically alleviate gout-related pain/bone damage. This transdermal platform enhances drug delivery efficiency, reduces systemic exposure, and demonstrates bone-protective effects through osteoclast inhibition and cartilage preservation.	In vivo (gouty rat arthritis model)	Li D. et al. (2024)
9		Colchicine	Borneol-modified ethosomes	Transdermal delivery	The anti-gout mechanism of borneol-modified colchicine ethosomes involves enhancing transdermal drug penetration to suppress MSU crystal-induced inflammation. This formulation reduces neutrophil activation, pro-inflammatory cytokine release, stabilizes blood levels, minimizes GI toxicity, and improves therapeutic efficacy.	In vivo (gouty rat arthritis model)	Zhang K. et al. (2019)
10		Alpha Phellandrene	Ethosomal Gel	Transdermal delivery	The anti-gout mechanism involves the ethosomal gel formulation of alpha-phellandrene suppressing inflammation by inhibiting cyclooxygenase-II, lipoxygenase-5, myeloperoxidase, and inducible nitric oxide synthase activities, thereby reducing pro-inflammatory mediator production.	In vitro study	Valsalan Soba et al. (2021)
11		Colchicine	Ethosomes cataplasm	Transdermal delivery	Inhibits inflammation caused by monosodium urate crystal deposition by suppressing pro-inflammatory cytokine release and reducing neutrophil recruitment, while minimizing systemic exposure.	In vivo (gouty rat arthritis model)	Wei et al. (2025)
12		Febuxostat	Self-emulsifying DDS	Oral delivery	The anti-gout mechanism involves the self-nanoemulsifying drug delivery system enhancing febuxostat's solubility and intestinal permeability, thereby improving its oral bioavailability and enabling sustained reduction of uric acid levels through efficient inhibition of xanthine oxidase activity.	In vivo (gouty rat arthritis model)	Rangaraj et al. (2019)
13		Febuxostat	Self-Nanoemulsifying DDS	Oral delivery	Febuxostat inhibits xanthine oxidase to lower uric acid synthesis, while SNELTs enhance its oral bioavailability (146.4% relative bioavailability) via nanosizing and lyophilization. This improves pharmacokinetics (↑Cmax, ↓Tmax, ↑AUC), enabling potent urate-lowering efficacy with reduced gastrointestinal side effects and dosing frequency.	ClinicalTrials	Al-Amodi et al. (2020)
14	Polymeric based DDS	Ginsenoside Rb1	Polymeric nanocapsules encapsulation	Oral delivery	Suppressed NF-κB signaling and NLRP3 inflammasome activation to reduce pro-inflammatory cytokines and mitochondrial damage, while nanoencapsulation enhanced drug targeting to inflamed joints, improving localized anti-inflammatory efficacy against MSU-induced gout.	In vivo (gouty rat arthritis model)	Liu J. et al. (2020)
15		15d-PGJ2	Polymeric nanocapsules	Injection	Suppressed NF-κB activation and NLRP3 inflammasome assembly, reducing MSU-induced IL-1β, TNF-α, IL-6, IL-17, and oxidative stress. Inhibited pro-inflammatory cytokine maturation in LPS-primed macrophages and joint inflammation via PPAR-γ-dependent pathways.	In vivo (gouty mice arthritis model)	Ruiz-Miyazawa et al. (2018)
16		Allopurinol	Nanospheres	Oral delivery	Allopurinol inhibits XO to lower uric acid levels, while niosomes enhance drug solubility, enable sustained release, and improve membrane interaction for prolonged therapeutic effects compared to free allopurinol.	In vivo (gouty rabbit arthritis model)	Singh et al. (2017)
17		PMN-Ecto	Neutrophil microvesicles	Injection	PMN-Ecto suppressed C5a-primed NLRP3 inflammasome activation via MerTK receptor signaling, reducing IL-1β release and neutrophil influx in MSU-induced inflammation. Concurrent TGF-β secretion was MerTK-independent but insufficient to resolve acute gout in vivo.	In vivo (gouty mice arthritis model)	Cumpelik et al. (2016)
18		Uricase and resveratrol	Multimodal smart systems	Injection/Irradiation	The anti-gout mechanism involves a biomimetic nanosystem that co-delivers uricase, Pt-HA/PDA nanozyme, and resveratrol to inflamed joints via M2 macrophage-exosome fusion membranes, enabling synergistic urate depletion, localized mild hyperthermia-induced tissue repair, and polarization of anti-inflammatory macrophages.	In vivo (gouty rat arthritis model)	Xu et al. (2024)
19		Capsaicin and thiocolchicoside	Nano-cubosomes	Transdermal delivery	The co-loaded nano-cubosomes enhance transdermal bioavailability of CAP and TCS, enabling synergistic anti-inflammatory effects via prostaglandin inhibition and analgesic activity through modulation of pain pathways. This dual action reduces inflammation and alleviates gout-associated symptoms.	In vivo (gouty rat arthritis model)	Khan et al. (2024)
20		Uricase and aceclofenac	polymeric nanoparticles	Transdermal delivery	Uricase degrades uric acid into allantoin, preventing urate crystal formation, while aceclofenac inhibits COX enzymes to reduce inflammation and pain. PLGA nanoparticles enhance localized drug delivery to joints, enabling sustained release and synergistic crystal dissolution/anti-inflammatory effects.	In vivo (gouty rabbit arthritis model)	Tiwari et al. (2015)
21		PDA@Pt	Multifunctional PDA@Pt nanomedicine	Injection	Synergistically degraded uric acid via Pt-catalyzed reactions, scavenged reactive oxygen species with polydopamine, and enhanced mitochondrial repair/anti-inflammatory effects through NIR-II photothermal therapy-mediated suppression of NF-κB signaling and pro-inflammatory cytokine release.	In vivo (gouty rat arthritis model)	Zhao et al. (2024)
22		BmK9 peptide and Uricase	BmK9-uricase nanoparticles	Intravenous	Nplex enables synergistic urate-lowering and anti-inflammatory effects (via BmK9-mediated cytokine modulation). The nanocomposite structure ensures sustained drug release, prolonged half-life, and enhanced tissue penetration, collectively reducing gouty inflammation and preventing urate crystal-induced nephropathy.	In vivo (gouty rat arthritis model)	Han et al. (2021)
23		IL-1Ra	chimera protein nanoparticles	Injection	The engineered nanoparticles extend IL-1Ra bioavailability via PEGylation-induced stabilization and electrostatic/hydrophobic interactions, enabling sustained IL-1β pathway blockade. This results in prolonged anti-inflammatory effects, reduced MSU crystal-driven inflammation, and extended dosing intervals in gouty arthritis.	In vivo (gouty rat arthritis model)	Zhang et al. (2021)
24		Pegloticase	PEG-drug conjugates	Intravenous	Pegloticase reduces serum urate levels through enzymatic urate degradation, while methotrexate's immunomodulatory effects suppress anti-drug antibody formation, thereby sustaining Pegloticase therapeutic efficacy, minimizing infusion-related reactions, and ultimately achieving sustained urate control alongside improvements in gout-related symptoms and patient quality of life.	ClinicalTrials.gov (NCT03635957)	Botson et al. (2022)
25		SEL-212	PEG-drug conjugates	Intravenous	SEL-212 synergizes PEGylated uricase-mediated urate degradation with sirolimus-induced immune tolerance, suppressing anti-drug antibody formation to sustain serum urate reduction and resolve tophi via long-term enzymatic activity.	ClinicalTrials.gov (NCT02959918)	Kivitz et al. (2023)
26		PEG-uricase	PEG-uricase	Intravenous	PEG-uricase catalyzes the enzymatic conversion of uric acid to soluble allantoin, rapidly reducing plasma urate levels and urinary urate excretion, thereby depleting tissue urate stores and suppressing crystal-driven inflammation in severe gout.	ClinicalTrials	Sundy et al. (2007)
27		Ibuprofen and diclofenac	Transdermal NSAID	Transdermal delivery	Gout Buster formulations enhance transdermal NSAID delivery (ibuprofen/diclofenac), promoting MSU crystal dissolution through synergistic mechanisms, increased drug penetration at inflammatory sites.	In vitro study	Sundy et al. (2007)
28		Luteolin	Composite nanofibers	Surgical implantation	Suppressed MSU-induced inflammation by inhibiting TNF-β, IL-1β, and IL-6 production in macrophages, blocked xanthine oxidase activity to reduce uric acid synthesis, and provided sustained drug release for long-term prevention of gout recurrence via localized implantation.	In vivo (gouty rabbit arthritis model)	Wang et al. (2016)
29		Febuxostat	HPMC gel	Transdermal delivery	FXT inhibits XO to lower uric acid levels, while the ethosomal system improves transdermal drug absorption, ensuring sustained release and enhanced therapeutic efficacy in gout management.	In vivo (gouty rat arthritis model)	El-Shenawy et al. (2019)
30		CMCS@SAG	Hydrogel microsphere	Injection	CMCS@SAG alleviates acute gouty arthritis through pH-responsive Gas6 release in acidic joint microenvironments, which suppresses macrophage-mediated inflammation, enhances mitochondrial resilience, and drives M2 macrophage polarization to resolve inflammatory infiltration and joint swelling.	In vivo (gouty mice arthritis model)	Chen Z. et al. (2025)
31		MCC950 /Entrectinib	Hybrid hydrogel	Cells culture	MCC950 and entrectinib suppress MSU crystal-induced NLRP3 inflammasome activation, modulate macrophage polarization toward anti-inflammatory phenotypes, and block neutrophil extravasation, thereby mitigating synovial inflammation in the chip model.	In vitro study	Lee et al. (2025)
32		Uricase and Dopamine	Explosive Hydrogel	Oral delivery	Intestinal microspheres exploit gut-specific triggers to immobilize uricase on mucosa, upregulate epithelial urate transporters via dopamine polymerization, and enhance fecal uric acid excretion by 30% while reducing serum urat and modulating gut microbiota in gout models.	In vivo (gouty mice arthritis model)	Tang et al. (2024)
33		Berberine	Composite nanogels	Intravenous	Ber-MAGN targets inflammatory joints via albumin-SPARC interaction, enabling localized and sustained berberine release to attenuate oxidative stress and suppress inflammatory responses in gouty arthritis.	In vivo (gouty rat arthritis model)	Sun et al. (2023)
34		Colchicine	Chitosan nanoparticle gel	Transdermal delivery	Colchicine inhibits microtubule polymerization to reduce neutrophil migration and inflammatory cytokine release, thereby alleviating gouty inflammation. The chitosan nanoparticle gel enhances transdermal penetration, sustaining local drug concentration for prolonged urate-lowering effects and improved joint histopathology.	In vivo (gouty rabbit arthritis model)	Parashar et al. (2022)
35		Colchicine	Hydrogel microneedle	Transdermal delivery	Col-HMNs enable efficient colchicine delivery via super-swelling, mechanically robust hydrogel matrices, sustaining local drug release to suppress acute gouty inflammation by downregulating pro-inflammatory cytokines (IL-1β, IL-6, TNF-α) and reducing neutrophil infiltration.	In vivo (gouty rat arthritis model)	Jiang et al. (2023)
36		Colchicine and uricase	Microneedles	Transdermal delivery	The system achieves sustained local delivery of colchicine (anti-inflammatory) and uricase (urate-degrading enzyme) over one week, reducing systemic drug exposure and avoiding daily oral colchicine use or high-dose uricase injections. It enhances therapeutic compliance by minimizing toxicity risks, stabilizing uricase.	In vivo (gouty rat arthritis model)	Yang B. et al. (2023)
37		Colchicine	Microneedles	Transdermal delivery	Separable silk fibroin microneedles enhance colchicine delivery through superior mechanical strength and skin insertion, reducing systemic drug exposure and irritation while sustaining local anti-inflammatory efficacy by suppressing cytokine cascades in acute gouty joints.	In vivo (gouty mice arthritis model)	Liao et al. (2023)
38		Febuxostat	Microneedles	Transdermal delivery	Cubosome-microneedle system enhances Febuxostat permeation through skin, improving oral bioavailability and reducing gastrointestinal side effects, thereby sustaining urate-lowering efficacy and suppressing inflammation in gouty joints via localized drug delivery.	In vivo (gouty rat arthritis model)	Patel and Thakkar (2023)
39		Colchicine	Microneedles	Transdermal delivery	Dissolvable Col-microneedles enable localized transdermal drug release, bypassing oral toxicity, to suppress acute gouty inflammation by reducing knee edema, mechanical hypernociception, and neutrophil infiltration in joints.	In vivo (gouty rat arthritis model)	Liu et al. (2022)
40		Oxypurinol	Microneedles	Transdermal delivery	TRG-PSMN enhances transdermal OXY delivery via microneedle-mediated skin penetration and sustained release, inhibiting xanthine oxidase to reduce serum urate levels while minimizing systemic toxicity and inflammation in gouty joints.	In vitro study	Mohamad et al. (2025)
41	Other DDS	Zinc ferrite nanoparticles	Low-Zn²⁺-doped zinc ferrite nanoparticles	Injection	ZFN mitigate gouty inflammation by modulating NF-κB signaling, suppressing NLRP3 inflammasome activation, and activating the Nrf2 antioxidant pathway, thereby reducing joint inflammation and oxidative stress.	In vivo (gouty mice arthritis model)	Zhang et al. (2025)
42		Sodium Salicylate	Polypyrrole bilayer structure	Transdermal delivery	The device enables localized, electrically controlled release of SSA through the skin, leveraging wireless power and flexibility. By adjusting the applied voltage, SSA release is precisely controlled for targeted gout therapy, minimizing systemic NSAID side effects.	In vitro study	Liu Y. et al. (2020)
43		Pt/CeO₂ nanozymes	Artificial organelles	-	Pt/CeO₂@Fe³⁺/FMPs catalyze self-cascade degradation of uric acid and simultaneous H₂O₂ scavenging, mimicking natural uricase/catalase activity to reduce urate levels and oxidative stress in gout.	In vitro study	You and Chen (2024)

**TABLE 2:** Summary of DDS treatment methods for hyperuricemia.

No	Type	Drug	Formulation	Route of administration	Anti-hyperuricemia mechanism	Clinical or preclinical trial	Reference
1	Lipid-based DDS	Sesquiterpene	Liposomes	Injection	Liposomal encapsulation enabled sustained release of eremantholide C and goyazensolide, preserving their xanthine oxidase inhibitory activity and ensuring prolonged reduction of serum uric acid levels	*In vivo* (hyperuricemic rat model)	Cunha Matosinhos et al. (2024)
2		Uricase	Liposomes	Intravenous	After encapsulating free uricase into multivesicular liposomes, the time to reach maximum plasma concentration is delayed, the half-life is prolonged, and bioavailability is significantly enhanced. This formulation enables more effective reduction of serum uric acid levels in hyperuricemic rat models	*In vivo* (hyperuricemic rat model)	Deng et al. (2015)
3		Sitogluside and dioscin	Liposomes	Oral delivery	P-SG-Dio significantly reduced the expression of pro-inflammatory cytokines IL-1β and IL-18 in serum and kidneys, modulated the NLRP3 inflammasome pathway, alleviated oxidative stress injury by decreasing serum malondialdehyde levels and enhancing superoxide dismutase and glutathione peroxidase activities while exhibiting no adverse effects on liver function indicators	*In vivo* (hyperuricemia mice model)	Liu W. et al. (2025)
4		Uricase	Liposomes	Oral delivery	PEGylated uricase encapsulated in liposomes protects the enzyme from gastrointestinal degradation, enhances intestinal absorption, and maintains catalytic activity in circulation to convert uric acid into excretable allantoin, thereby reducing serum uric acid levels	*In vivo* (chicken model)	Nishida et al. (1984)
5		Uricase	Biomimetic vesicles	Intravenous	These biomimetic nanovesicles/microassemblies as enzyme carriers enhance enzymatic activity, prolong action duration, and boost therapeutic efficacy. *In vivo* studies demonstrate significantly improved bioavailability (approximately 533% and 331% increases vs native uricase) and superior treatment outcomes, with both UHLN and UHLNM exhibiting superior uricase delivery properties	*In vivo* (hyperuricemic rat model)	Yang et al. (2020)
6		Myricitrin	Proliposomes	Oral delivery	MPs enhance myricetin’s therapeutic efficacy by reducing serum uric acid levels. Histopathological analysis further confirms that MPs significantly strengthen myricetin’s hepatorenal protective effects in hyperuricemic rats	*In vivo* (hyperuricemic rat model)	Weng et al. (2019)
7		[6]-Shogaol	Solid lipid nanoparticles	Oral delivery	SLNs significantly enhanced [6]-shogaol’s solubility and oral bioavailability, demonstrating superior efficacy in hyperuricemic/gouty arthritis models by markedly reducing serum uric acid, IL-1β, and TNF-α levels compared to free [6]-shogaol. Mechanistically, SLNs synergized [6]-shogaol’s xanthine oxidase inhibition with augmented anti-inflammatory effects while attenuating multi-organ damage	*In vivo* (hyperuricemic rat model)	Wang H. et al. (2018)
8		Liquiritin	Self-emulsifying DDS	Oral delivery	LQ–SNEDDS enhances liquiritin’s anti-hyperuricemic efficacy by improving dissolution/bioavailability, inhibiting uric acid synthesis, accelerating drug release, and mitigating multi-organ damage, thereby significantly boosting its therapeutic potential against hyperuricemia	*In vivo* (hyperuricemic rat model)	Wei et al. (2022)
9		Morin–phospholipid	Self-microemulsifying DDS	Oral delivery	Morin–phospholipid, formulated through self-microemulsifying drug delivery system, enhances anti-hyperuricemic efficacy via three mechanisms, MPC–SNEDDS significantly elevates maclurin accumulation in liver/kidneys with prolonged retention, dual inhibition of hepatic XDH/XO at mRNA and enzymatic levels reduces uric acid production, and restoration of renal transporter dysregulation promotes uric acid excretion	*In vivo* (hyperuricemic rat model)	Zhang et al. (2016)
10		6-Shogaol	Self-microemulsifying DDS	Oral delivery	6-Shogaol–SMEDDS significantly enhances solubility/absorption, achieving 571.18% increased oral bioavailability, inhibits xanthine oxidase to reduce serum uric acid (71.08% decline at 100 mg/kg), and mitigates renal damage (e.g., cytoplasmic vacuolation and inflammation) while selectively accumulating in the liver/kidneys for targeted anti-hyperuricemic efficacy	*In vivo* (hyperuricemic rat model)	Yang M. et al. (2019)
11		Isoliquiritigenin	Self-microemulsifying DDS	Oral delivery	The ISL–SMEDDS formulation, composed of ethyl oleate, Tween 80, and PEG 400, significantly enhances oral bioavailability and accelerates drug release, thereby reducing serum uric acid levels in hyperuricemic rats through xanthine oxidase inhibition and by improving therapeutic efficacy via targeted delivery	*In vivo* (hyperuricemic rat model)	Zhang Y. et al. (2019)
12		Licochalcone A	Self-microemulsifying DDS	Oral delivery	LCA–SMEDDS enhances licochalcone A’s oral bioavailability by 2.36-fold through improved solubility and absorption, thereby significantly reducing serum uric acid levels (60.08% decrease) in hyperuricemic rats via efficient drug delivery and targeted therapeutic action	*In vivo* (hyperuricemic rat model)	Zhu et al. (2021)
13	Polymeric based DDS	Scopoletin	Polymeric micelles	Oral delivery	Soluplus micelles significantly enhances scopoletin’s oral bioavailability (4.38-fold AUC increase) and intestinal absorption, particularly in the duodenum/jejunum, thereby effectively reducing serum uric acid to normal levels in hyperuricemic mice via improved drug solubilization and targeted hepatic accumulation	*In vivo* (hyperuricemic mice model)	Zeng et al. (2017)
14		Scopoletin	Polymeric micelles	Oral delivery	Sco-Ms enhance scopoletin’s anti-hyperuricemic efficacy by improving oral bioavailability (438% increase) and dual-modulating urate metabolism, upregulating renal URAT1/GLUT9/OAT1 expression to promote excretion, and suppressing hepatic XOD activity to reduce production, thereby normalizing serum uric acid levels and attenuating renal damage more effectively than free scopoletin	*In vivo* (hyperuricemic mice model)	Zeng et al. (2020)
15		Pinocembrin	Polymeric micelles	Oral delivery	PCB-FPM, a novel polymeric micelle system, significantly enhances pinocembrin’s solubility, oral bioavailability (2.61-fold increase), and anti-hyperuricemic efficacy by 78.82% UA reduction in rats, offering a promising strategy for improving therapeutic outcomes against hyperuricemia	*In vivo* (hyperuricemic rat model)	
16		ImmTOR	Nanocapsules	Intravenous	Clinical trials demonstrate that ImmTOR, a biodegradable nanoparticle encapsulating rapamycin, when co-administered with pegadricase, a pegylated uricase enzyme, effectively mitigates immunogenicity and enhances therapeutic efficacy in hyperuricemia patients. ImmTOR significantly inhibits the formation of anti-drug antibodies against pegadricase in a dose-dependent manner, thereby sustaining enzyme activity and reducing serum uric acid levels	ClinicalTrials.gov (NCT02464605) (NCT02648269)	Sands et al. (2022)
17		Urate oxidase	Nanocapsules	Intravenous	Poly (N-vinylpyrrolidone)-encapsulated urate oxidase nanocapsules enhance anti-hyperuricemic efficacy by improving protein stability, prolonging systemic circulation (>10-fold), and reducing macrophage clearance, thereby enabling sustained enzymatic degradation of uric acid and effective management of hyperuricemia while providing a versatile platform for broader protein therapeutic applications	*In vivo* (hyperuricemic mice model)	Zhang et al. (2017)
18		Uricase	Nanocapsules	Injection	Uricase nanocapsule assemblies enhance anti-hyperuricemic efficacy by protecting encapsulated uricase from rapid bloodstream clearance, thereby sustaining catalytic activity and enabling prolonged uric acid degradation. They improve bioavailability, reduce immunogenicity, and induce favorable conformational changes in uricase, demonstrating superior therapeutic performance	*In vivo* (hyperuricemic rat model)	Xiong et al. (2016)
19		Allopurinol	Nanoparticles	Intravenous	Allopurinol-loaded bovine serum albumin nanoparticles enhance anti-hyperuricemic efficacy by selectively targeting kidneys through albumin–receptor interactions, thereby increasing renal drug accumulation, reducing serum uric acid levels, and mitigating nephrolithiasis via localized therapeutic action	*In vivo* (hyperuricemic mice model)	Kandav et al. (2020)
20		Allopurinol	Nanoparticles	Intravenous	Chitosan nanoparticles formulated with low-molecular-weight chitosan enhance anti-hyperuricemic efficacy by selectively targeting kidneys via pH-sensitive drug release and improved renal accumulation, thereby reducing serum/urine uric acid levels and urine pH to effectively manage hyperuricemic nephrolithiasis through localized therapeutic action and enhanced drug bioavailability	*In vivo* (hyperuricemic mice model)	Kandav et al. (2022)
21		Urate oxidase–albumin conjugate	Hydrogels	Injection	Injectable hydrogels leveraging strong albumin-binding peptide–human serum albumin interactions enable sustained release of urate oxidase, significantly extending its serum half-life and maintaining therapeutic efficacy in hyperuricemia by continuously degrading uric acid	*In vivo* (hyperuricemic mice model)	Cho et al. (2020)
22		Allopurinol	Microneedles	Transdermal delivery	Allopurinol-loaded dissolving microneedles enhance anti-hyperuricemic efficacy by enabling sustained transdermal drug release, circumventing hepatic first-pass metabolism, and improving bioavailability, thereby reducing adverse effects while maintaining therapeutic urate-lowering effects through localized cutaneous drug reservoirs	*In vivo* (hyperuricemic mice model)	Chen et al. (2021)
23		Allopurinol	Microneedles	Transdermal delivery	A core-shell microneedle patch synergistically reduces hyperuricemia through immediate allopurinol release to inhibit uric acid synthesis and sustained urate oxidase-mediated degradation coupled with calcium peroxide-driven oxidation, achieving rapid normalization of serum uric acid within 3 h and sustained hypouricemic control for 12 h while mitigating hepatorenal toxicity by stabilizing renal biomarkers and suppressing hepatic xanthine oxidase activity	*In vivo* (hyperuricemic mice model)	Wang X. et al. (2024)
24		Allopurinol	Microneedles	Transdermal delivery	A polymer microneedle system combining polyvinylpyrrolidone (PVP) and polycaprolactone (PCL) enables controlled transdermal allopurinol delivery, achieving rapid hypouricemic effects through PVP-mediated burst release, followed by sustained drug release via PCL degradation, thereby reducing serum uric acid levels with fewer adverse effects than oral administration in hyperuricemic mice	*In vivo* (hyperuricemic mice model)	Wang et al. (2023)
25		Uricase and HRP	Microneedles	Transdermal delivery	A novel uricase and HRP–CaHPO_4_ nanoflower-integrated hyaluronic acid microneedle system enhances anti-hyperuricemic efficacy by transdermal delivery of stabilized enzymes, where CaHPO_4_ nanoflowers improve uricase stability/activity and HRP accelerates uric acid oxidation via peroxide decomposition, achieving sustained blood urate reduction comparable to intravenous injection with minimal adverse effects	*In vivo* (hyperuricemic mice model)	Hao et al. (2019)
26	Other DDSs	Deinococcus radiodurans-derived protein	Synthetic gene network	Intraperitoneal implantation	A synthetic gene circuit integrating a uric acid-responsive Deinococcus radiodurans protein and secreted Aspergillus flavus urate oxidase maintains urate homeostasis by triggering dose-dependent enzymatic degradation of uric acid, thereby reducing blood urate levels and renal crystal deposition in hyperuricemic mice through a self-regulating metabolic feedback mechanism	*In vivo* (urate oxidase-deficient mice)	Kemmer et al. (2010)
27		Uri/Cat@ArtPC	Artificial organelles	Intravenous	Artificial protocells composed of polylysine–polynucleotide coacervate droplets encapsulate a uricase–catalase catalytic system to degrade uric acid and detoxify H_2_O_2_, achieving sustained hypouricemic effects by maintaining serum urate homeostasis and preventing renal injury in hyperuricemic mice through localized enzymatic catalysis and enhanced biocompatibility	*In vivo* (hyperuricemic mice model)	Hu Q. et al. (2024)
28		UOX-CAT@ZIF-8-RBC	ZIF-8 framework-based nanoparticles	Intravenous	Armored red blood cell biohybrids encapsulating ZIF-8-protected urate oxidase and catalase achieve rapid and sustained anti-hyperuricemic effects by combining extended circulatory persistence with enzymatic degradation of uric acid and neutralization of toxic H_2_O_2_, normalizing serum urate levels	*In vivo* (hyperuricemic mice model)	Li Z. et al. (2024)
29		UOX-AuNP@NCs	Biocompatible pluronic-based nanocarriers	Intravenous	A biocompatible pluronic-based nanocarrier co-delivering urate oxidase and catalase-mimic gold nanoparticles enhances hyperuricemia treatment by synergistically degrading uric acid while neutralizing cytotoxic H_2_O_2_ by-products, thereby reducing oxidative toxicity and improving therapeutic efficacy compared to free enzyme formulations in both cellular and murine models	*In vivo* (hyperuricemic mice model)	Kim et al. (2019)

### 4.3 Polymeric-based DDSs

Polymeric-based DDSs represent a class of platforms that utilize polymer materials as carriers to enhance drug delivery, playing a pivotal role in the fields of pharmacy, medicine, and healthcare. These systems achieve controlled drug release by modulating the physicochemical properties of polymers, such as solubility, release kinetics, and targeting capabilities, thereby enhancing therapeutic efficacy and reducing adverse effects (Gonzalez et al., 2023). Polymers, which can be natural or synthetic [e.g., chitosan, poly (lactic acid) (PLA), poly (glycolic acid) (PGA)], exhibit excellent biocompatibility and biodegradability, making them suitable for various delivery routes (Harun-Or-Rashid et al., 2023). In the context of gout and hyperuricemia treatment, we categorize these systems into basic polymeric nanocarrier systems, functionalized and composite polymeric carrier systems, hydrogel and fibrous polymeric carrier systems, and microneedle delivery systems.

#### 4.3.1 Basic polymeric nanocarrier systems

Nanoparticle systems use polymeric nanoparticles as core carriers to effectively encapsulate drugs and deliver them to specific sites (e.g., joints and tumor regions) through passive or active targeting mechanisms (Zhao and Zhou, 2022). This design facilitates precise therapeutic delivery, improving bioavailability and reducing systemic toxicity. Common formulations include polymeric nanocapsules, nanospheres, and polymeric nanoparticles, which are utilized in oral or transdermal delivery systems to enhance drug absorption (Singh et al., 2017; Tiwari et al., 2015). Basic polymeric nanocarrier systems significantly enhance the therapeutic efficacy in gout treatment through multi-pathway synergistic mechanisms. For instance, ginsenoside Rb1 nanocapsules orally target inflamed joints, inhibiting the NF-κB/NLRP3 pathway and mitochondrial damage (Liu J. et al., 2020); 15d-PGJ2 nanoformulations block the maturation of various pro-inflammatory cytokines via PPAR-γ-dependent pathways (Ruiz-Miyazawa et al., 2018); and intelligent biomimetic nanosystems combine uricase degradation, near-infrared hyperthermia, and M2 macrophage membrane targeting to achieve dual interventions in uric acid metabolism regulation and inflammatory repair (Xu et al., 2024). These carriers optimize drug delivery efficiency, prolong action duration, and activate multi-target anti-inflammatory pathways, demonstrating superior local efficacy and symptomatic relief compared to conventional administration in rodent and rabbit gout models.

Similarly, basic polymeric nanocarrier systems significantly optimize the therapeutic effects in hyperuricemia through multi-mechanism synergies. Scopoletin and pinocembrin, when encapsulated in polymeric micelles, exhibit urate-lowering effects by inhibiting xanthine oxidase activity and promoting uric acid excretion, respectively, while significantly improving renal injury protection (Zeng et al., 2020; Rong et al., 2022; Zeng et al., 2017). Uricase nanocapsules extend the catalytic activity cycle of the enzyme, converting uric acid into readily excretable allantoin, whereas allopurinol nanoformulations enhance the inhibition of uric acid synthesis and the prevention of kidney stones through targeted delivery (Kandav et al., 2020; Xiong et al., 2016). Furthermore, these nanosystems prolong uricase efficacy through immunomodulation, collectively highlighting the pivotal role of nanocarriers in enhancing drug bioavailability, targeting, and multi-pathway synergistic therapy.

#### 4.3.2 Functionalized and composite polymeric carrier systems

Polymeric carriers are often engineered through surface modification or multifunctional design to enable drug loading, controlled release, and targeted delivery, thereby enhancing therapeutic efficacy while minimizing toxicity and adverse effects (Lima and Reis, 2021). These systems manifest in diverse forms, including nanoparticles, conjugates, and composite systems, offering advantages such as high drug-loading capacity, sustained release profiles, and reduced immunogenicity (Gulyaev et al., 2025). For instance, PDA–PEG and PDA–PEG–TPP nanoparticles (where TPP denotes triphenylphosphine) facilitate targeted delivery to the nucleus and mitochondria, enhancing drug accumulation in cancer cells and overcoming drug resistance (Li et al., 2017). Studies demonstrate that PDA–PEG–TPP–DOX exhibits superior mitochondrial targeting capability both *in vitro* and *in vivo*. Furthermore, these systems synergistically degrade uric acid via platinum-catalyzed reactions, scavenge ROS using polydopamine, and enhance mitochondrial repair/anti-inflammatory effects through near-infrared-II photothermal therapy-mediated suppression of NF-κB signaling and pro-inflammatory cytokine release (Zhao et al., 2024).

The BmK9–uricase nanoparticle represents a composite carrier system based on electrostatic complexation between neutral scorpion toxin BmK9 and uricase. This Nplex platform enables synergistic urate-lowering and anti-inflammatory effects via BmK9-mediated cytokine modulation. The nanocomposite architecture ensures sustained drug release, prolonged half-life, and enhanced tissue penetration, collectively mitigating gouty inflammation and preventing urate crystal-induced nephropathy (Han et al., 2021).

Additionally, PEG–drug conjugates are prevalent functionalized carriers where PEG modifies therapeutic agents (e.g., uricase) to improve pharmacokinetics and reduce immunogenicity (Ozer et al., 2022). For example, uricase–PEG conjugates diminish immunogenicity and extend half-life. Similarly, PEGylated IL-1Ra chimeric protein nanoparticles prolong half-life, enabling sustained blockade of the IL-1β pathway and inhibition of MSU crystal-induced inflammation. In clinical settings, pegloticase and SEL-212 formulations, combined with immunomodulators (e.g., methotrexate/sirolimus), suppress anti-drug antibody formation to maintain uricase activity, achieving long-term uric acid control and tophus resolution. Furthermore, sodium-containing formulations should be judiciously avoided in topical preparations, while fully optimized delivery systems are indispensable for enhancing transdermal NSAID penetration, enabling precise targeting to inflammatory foci, and achieving synergistic therapeutic outcomes through dual anti-inflammatory and crystal-dissolving mechanisms (Hooper and He, 2022). By extending drug action cycles, inducing immune tolerance, and activating multi-target synergistic mechanisms, these carrier systems offer highly efficient and safe delivery strategies for gout management.

#### 4.3.3 Hydrogel and fibrous polymeric carrier systems

Hydrogel and fibrous polymeric carrier systems enable precise targeting and multi-mechanistic synergies in gout therapeutics through localized delivery, sustained release, and environmental responsiveness, thereby enhancing efficacy while reducing adverse effects. For instance, composite nanofibers composed of gelatin, chitosan, collagen, or sodium alginate can be fabricated via electrospinning to create porous scaffolds that mimic the extracellular matrix, promoting cell adhesion and proliferation (e.g., spindle-shaped human dermal fibroblasts on gelatin/chitosan scaffolds) (Pezeshki-Modaress et al., 2018). Electrospun poly (lactic-co-glycolic acid)/gelatin composite fibers loaded with luteolin achieve sustained intra-articular release, suppressing MSU-induced TNF-α, IL-1β, and IL-6 production while inhibiting xanthine oxidase activity for long-term recurrence prevention in rabbit models (Wang et al., 2016).

The fundamental attributes of hydrogels lie in their three-dimensional hydrophilic polymer networks, which exhibit biocompatibility, biodegradability, and high drug-loading capacity, enabling on-demand drug release in response to environmental stimuli (e.g., pH and temperature) (Mathew et al., 2018). Transdermal febuxostat-loaded hydroxypropyl methylcellulose hydrogels enhance drug permeability via ethosomal formulations, enabling sustained urate-lowering effects in gouty rats (El-Shenawy et al., 2019). pH-responsive CMCS@SAG hydrogel microspheres selectively release Gas6 in acidic joint microenvironments, promoting M2 macrophage polarization and mitochondrial stabilization to resolve acute gouty inflammation (Chen F. et al., 2025). Additionally, PEG–PAEU–ABP hybrid hydrogels loaded with uricase–human serum albumin effectively stabilize serum uric acid levels in hyperuricemic mice, offering prolonged nephron protection against hyperuricemic damage through extended and sustained release mechanisms (Cho et al., 2020). Collectively, these systems exemplify spatially controlled drug release, microenvironment-responsive activation, and combinatorial anti-inflammatory/urate-regulating strategies for advanced gout therapy.

#### 4.3.4 Microneedle delivery systems

Microneedles represent a minimally invasive drug delivery platform composed of microscale needles (height: 10–2000 μm; width: 10–50 µm) designed to penetrate the stratum corneum barrier and create transient microchannels for painless, transdermal drug administration into the dermis (Hao et al., 2017). Classified by morphology and drug release mechanisms, microneedle variants include solid, coated, dissolving, hollow, and hydrogel-forming types (Kumar and Shukla, 2024). For example, solid microneedles use a “poke-and-patch” strategy, whereas dissolving microneedles directly release encapsulated drugs upon dissolution within the skin (He et al., 2019). Microneedle technology offers advantages such as enhanced bioavailability, reduced pain, improved patient compliance, and ease of operation compared to conventional injections (Zhao et al., 2020). Microneedle delivery systems have emerged as transformative tools for gout therapy, enabling localized and sustained drug release while minimizing systemic toxicity. Colchicine-loaded hydrogel microneedles demonstrate prolonged anti-inflammatory efficacy by downregulating pro-inflammatory cytokines (IL-1β, IL-6, and TNF-α) and reducing neutrophil infiltration in gouty joints, as evidenced in rat arthritis models (Jiang et al., 2023). Dissolvable silk fibroin microneedles enhance drug delivery efficiency through robust mechanical penetration, ensuring targeted suppression of cytokine cascades and edema in acute gouty inflammation, with reduced systemic exposure compared to oral administration (Liao et al., 2023).

Microneedle-based platforms address critical limitations of conventional oral therapies for hyperuricemia, such as hepatic first-pass metabolism and poor bioavailability. Transdermal microneedles loaded with allopurinol achieve sustained urate-lowering effects by controlling drug release kinetics while minimizing gastrointestinal side effects observed with oral formulations (Chen et al., 2021). Advanced systems integrating uricase and horseradish peroxidase within CaHPO_4_ nanoflowers, coupled with hyaluronic acid microneedles, enhance enzyme stability and catalytic activity, effectively reducing serum uric acid levels by 30% in murine models (Hao et al., 2019). Collectively, these platforms underscore the potential of microneedles to improve treatment adherence, efficacy, and safety in hyperuricemia management.

Polymeric drug delivery systems (PDDS) offer transformative potential for gout and hyperuricemia through controlled release, targeted delivery, and multi-mechanistic synergies. The specific details of this part can be found in Tables 1 and 2, which provide a comprehensive overview of the application of DDSs in the treatment of gout and hyperuricemia for this category. However, critical barriers impede clinical translation. (1) Regulatory and scalability gaps: standardized protocols for polymeric carrier scalability, sterilization, and long-term stability are lacking, with advanced systems (e.g., PEGylated uricase and biomimetic nanosystems) requiring rigorous immunogenicity (anti-drug antibodies) and cost-effectiveness evaluations. (2) Preclinical–clinical disconnect: dosing regimens (e.g., colchicine microneedles) lack human PK/PD validation, while transdermal hydrogels need human skin permeability studies to address species-specific stratum corneum variability. (3) Technical limitations: polymeric nanocarriers suffer from premature drug leakage; hydrogels require mechanical strength optimization; and microneedles lack large-scale manufacturing validation and patient compliance data. Future efforts must prioritize multi-center trials for safety/PK profiling, biodegradable polymers with tunable degradation, and multi-omics biomarkers for personalized therapy. Bridging these gaps will facilitate the adoption of PDDS in precision gout management.

### 4.4 Other DDSs

Beyond conventional nanocarriers, emerging DDS platforms demonstrate unique advantages in managing gout and hyperuricemia through tailored mechanisms. For gout, low-Zn^2+^-doped zinc ferrite nanoparticles exhibit dual anti-inflammatory and antioxidant effects by modulating NF-κB/NLRP3 signaling and activating the Nrf2 pathway, thereby reducing joint inflammation and oxidative stress in murine models (Zhang et al., 2025). Electrically responsive polypyrrole bilayer devices enable precise, wireless-controlled transdermal delivery of sodium salicylate, minimizing systemic NSAID toxicity while maintaining localized anti-inflammatory efficacy (Liu Y. et al., 2020). Additionally, Pt/CeO_2_ nanozymes mimic natural uricase/catalase activity through self-cascade uric acid degradation and H_2_O_2_ scavenging, offering a biomimetic approach to mitigate urate crystal-induced oxidative damage *in vitro* (You and Chen, 2024).

In hyperuricemia management, synthetic gene networks integrating HucR-based biosensors and secreted Aspergillus flavus urate oxidase achieve self-regulated urate degradation, dynamically adjusting enzyme expression to maintain physiological UA levels while avoiding excessive urate depletion (Kemmer et al., 2010). Artificial organelles (Uri/Cat@ArtPC) leverage ε-polylysine/DNA phase separation to co-encapsulate uricase and catalase, enabling synergistic urate conversion and H_2_O_2_ neutralization, with enhanced renal targeting and catalytic durability (Hu H. et al., 2024). These platforms collectively advance therapeutic precision by integrating biosensing, self-regulated enzyme activity, and biomimetic catalysis, addressing critical limitations of traditional urate-lowering therapies. However, due to the overlapping and distinct mechanisms underlying DDS applications in gout and hyperuricemia, published research to date provides comprehensive analyses, as summarized in Tables 1 and 2.

Emerging DDSs for gout and hyperuricemia, including zinc ferrite nanoparticles and synthetic gene networks, demonstrate innovative mechanisms such as self-regulated enzyme activity and biomimetic catalysis. Tables 1, 2 elaborate on the detailed application of the DDS in gout and hyperuricemia, respectively. However, clinical translation is hindered by regulatory/scalability challenges, cost barriers, preclinical–clinical translation gaps, and technical limitations (e.g., wireless control precision and *in vivo* stability). Future priorities include optimizing biodegradability, conducting pilot trials, and integrating multi-omics data to advance precision therapy.

### 4.5 Artificial intelligence DDS platform

Although AI-based DDSs remain underexplored in gout and hyperuricemia management, their transformative potential is evident. By integrating bioinformatics data with machine learning algorithms, AI can accelerate target discovery, predict pharmacokinetic profiles, and construct intelligent databases for real-time analysis of multi-omics data, thereby enabling personalized delivery strategies (Hassanzadeh et al., 2019). Key advantages include optimizing nanorobot design, predicting trans-biological barrier behavior, and dynamically adjusting delivery parameters via real-time feedback loops (Rai et al., 2023). Future directions may involve developing urate-sensitive smart carriers for targeted renal or joint delivery, utilizing pH-responsive/enzyme-triggered mechanisms to achieve spatiotemporal control of urate-lowering agents. Additionally, AI-driven disease progression models could optimize delivery timing, while metabolic profiling data may guide the development of systems targeting renal urate transporters (e.g., mURAT1), enhancing intervention precision for chronic metabolic disorders. These innovations hold promise to elevate therapeutic efficacy and patient-specific outcomes in gout management.

## 5 Conclusion and prospect

In conclusion, this review underscores the revolutionary potential of DDSs in transforming gout and hyperuricemia management through enhanced precision, efficacy, and patient compliance. Modern DDSs—including lipid-based, polymeric, and stimuli-responsive platforms—have demonstrated superiority over conventional therapies by optimizing pharmacokinetics, minimizing off-target effects, and enabling targeted delivery to pathological sites (e.g., renal tubules and inflamed joints). Notable advancements, such as uricase-loaded nanocarriers with reduced immunogenicity and pH-responsive hydrogels for sustained urate control, exemplify the shift toward intelligent, patient-centric designs.

However, critical limitations must be acknowledged. First, clinical validation remains inadequate: although preclinical data highlight the promise of multimodal therapies (e.g., co-delivering urate-lowering agents and anti-inflammatory drugs), robust clinical evidence is lacking. Second, safety and ethical concerns persist, particularly for injectable systems such as PEGylated uricase, where immunogenicity and long-term tissue accumulation necessitate rigorous risk–benefit analyses. Third, technical barriers—such as the scalability of nanofabrication processes, the cost-effectiveness of advanced materials, and heterogeneity in patient response—hinder translational progress.

To address these gaps, future research should prioritize the following. (1) Multifunctional DDS development: integrating real-time monitoring (e.g., biosensors for urate levels) with adaptive release mechanisms to enable personalized dose adjustment. (2) Preclinical-to-clinical translation: conducting phase I/II trials to validate efficacy, safety, and pharmacokinetic profiles of emerging platforms (e.g., artificial organelles and nanozymes). (3) Ethical and regulatory frameworks: establishing guidelines for immunogenicity testing, long-term safety surveillance, and patient consent protocols for experimental therapies. (4) Interdisciplinary collaboration: leveraging AI and omics technologies to predict therapeutic outcomes, optimize material properties, and identify biomarkers for stratified patient populations.

The future of DDSs for gout and hyperuricemia hinges on three interconnected directions. First, the integration of advanced biomaterials with artificial intelligence could enable self-regulating systems capable of dynamic dose adjustment based on individual metabolic fluctuations. Second, multimodal therapies combining urate-lowering agents, anti-inflammatory cytokines, and immunomodulators within a single platform may synergize to halt disease progression while minimizing systemic side effects. Third, decentralized manufacturing technologies, such as 3D bioprinting and microfluidic synthesis, could democratize access to customized DDSs, particularly in resource-limited settings.

The evolution of DDSs from passive carriers to intelligent, patient-centric platforms signifies promising progress in gout and hyperuricemia management, yet substantial challenges in clinical validation, scalability, and long-term safety persist, underscoring the need for sustained interdisciplinary efforts to fully realize their therapeutic potential. Realizing this potential requires addressing critical gaps: although preclinical studies highlight innovative mechanisms (e.g., multimodal therapies and stimuli-responsive release), robust clinical validation remains scarce, necessitating phase I/II trials to confirm efficacy and safety. Ethical and technical challenges—such as immunogenicity risks in injectable formulations (e.g., PEGylated uricase), long-term biocompatibility of advanced materials, and scalability of nanofabrication processes—demand rigorous surveillance frameworks and cost-effective manufacturing strategies. Ultimately, the field’s progression hinges on interdisciplinary collaboration to integrate adaptive technologies (e.g., AI-driven real-time monitoring), establish standardized safety protocols, and prioritize patient-centered design. By confronting these barriers, DDS can transition from conceptual breakthroughs to clinically validated solutions, offering safer, more equitable, and globally accessible therapeutic strategies to reduce the socioeconomic burden of gout and hyperuricemia.
